# Isl1 Directly Controls a Cholinergic Neuronal Identity in the Developing Forebrain and Spinal Cord by Forming Cell Type-Specific Complexes

**DOI:** 10.1371/journal.pgen.1004280

**Published:** 2014-04-24

**Authors:** Hyong-Ho Cho, Francesca Cargnin, Yujin Kim, Bora Lee, Ryuk-Jun Kwon, Heejin Nam, Rongkun Shen, Anthony P. Barnes, Jae W. Lee, Seunghee Lee, Soo-Kyung Lee

**Affiliations:** 1 Pediatric Neuroscience Research Program, Papé Family Pediatric Research Institute, Department of Pediatrics, Portland, Oregon, United States of America; 2 Department of Otolaryngology–Head and Neck Surgery, Chonnam National University Medical School, Gwangju, Korea; 3 College of Pharmacy and Research Institute of Pharmaceutical Sciences, Seoul National University, Seoul, Korea; 4 Vollum Institute, Oregon Health & Science University, Portland, Oregon, United States of America; 5 Department of Cell and Developmental Biology, Oregon Health & Science University, Portland, Oregon, United States of America; University of California Los Angeles, United States of America

## Abstract

The establishment of correct neurotransmitter characteristics is an essential step of neuronal fate specification in CNS development. However, very little is known about how a battery of genes involved in the determination of a specific type of chemical-driven neurotransmission is coordinately regulated during vertebrate development. Here, we investigated the gene regulatory networks that specify the cholinergic neuronal fates in the spinal cord and forebrain, specifically, spinal motor neurons (MNs) and forebrain cholinergic neurons (FCNs). Conditional inactivation of *Isl1*, a LIM homeodomain factor expressed in both differentiating MNs and FCNs, led to a drastic loss of cholinergic neurons in the developing spinal cord and forebrain. We found that Isl1 forms two related, but distinct types of complexes, the Isl1-Lhx3-hexamer in MNs and the Isl1-Lhx8-hexamer in FCNs. Interestingly, our genome-wide ChIP-seq analysis revealed that the Isl1-Lhx3-hexamer binds to a suite of cholinergic pathway genes encoding the core constituents of the cholinergic neurotransmission system, such as acetylcholine synthesizing enzymes and transporters. Consistently, the Isl1-Lhx3-hexamer directly coordinated upregulation of cholinergic pathways genes in embryonic spinal cord. Similarly, in the developing forebrain, the Isl1-Lhx8-hexamer was recruited to the cholinergic gene battery and promoted cholinergic gene expression. Furthermore, the expression of the Isl1-Lhx8-complex enabled the acquisition of cholinergic fate in embryonic stem cell-derived neurons. Together, our studies show a shared molecular mechanism that determines the cholinergic neuronal fate in the spinal cord and forebrain, and uncover an important gene regulatory mechanism that directs a specific neurotransmitter identity in vertebrate CNS development.

## Author Summary

Neurons utilize various chemicals to transmit signals to a target cell. Distinct types of neurons in the spinal cord and forebrain, collectively termed cholinergic neurons, utilize the same chemical, acetylcholine, for signal transmission. These neurons play critical roles in controlling locomotion and cognition. In this study, we have found that the *Isl1* gene orchestrates the process to generate cholinergic neurons in the spinal cord and forebrain. Isl1 forms two different types of multi-protein complexes in the spinal cord and forebrain. Both complexes bind the same genomic regions in a group of genes critical for cholinergic signal transmission, and promote their simultaneous expression. These cholinergic genes include enzymes that synthesize acetylcholine and proteins required to package acetylcholine into vesicles. The Isl1-containing multi-protein complexes were able to trigger the generation of cholinergic neurons in embryonic stem cells and neural stem cells. Our study reveals crucial mechanisms to coordinate the expression of genes in the same biological pathway in different cell types. Furthermore, it suggests a new strategy to produce cholinergic neurons from stem cells.

## Introduction

The choice of neurotransmitter is one of the most fundamental aspects of neuronal fate decision. Cholinergic neurons are located in diverse regions of the CNS, which do not share the developmental origin, and regulate complex behaviors. In the spinal cord, cholinergic motor neurons (MNs) control locomotion, whereas in the forebrain, cholinergic neurons regulate cognitive processes [1], [2]. Defects in function or survival of cholinergic neurons result in severe human pathologies, including spinal cord injuries, diseases associated with impaired motor function and cognitive disorders resulting from the loss of forebrain cholinergic neurons (FCNs) [3]. Despite the crucial roles of cholinergic neurons in human physiology and pathology, the mechanisms that specify cholinergic neuronal cell fate throughout the CNS during vertebrate development remain largely unknown.

**Figure 1:**
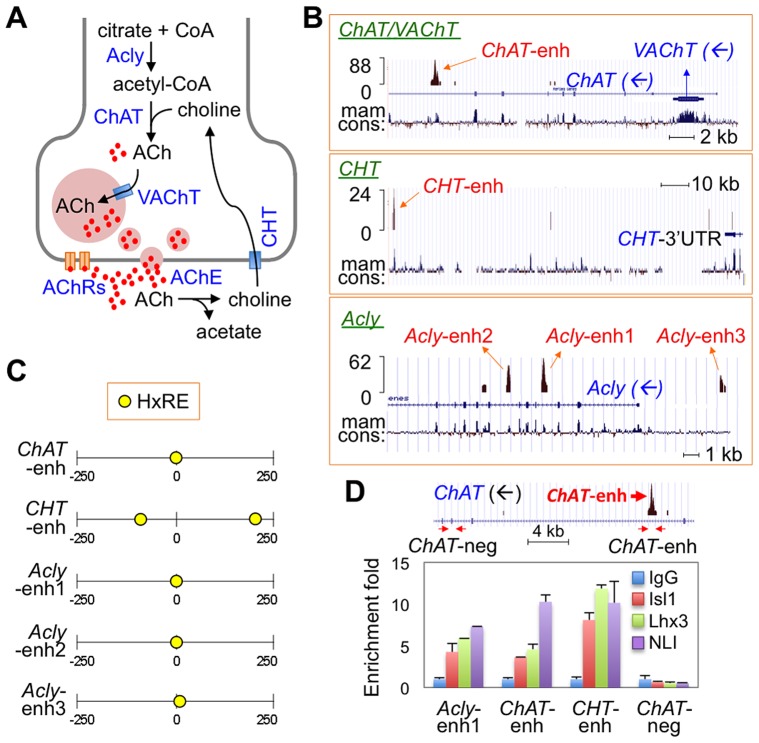
ChIP-seq assays revealed Isl-Lhx3-hexamer-binding sites in a cholinergic gene battery. (**A**) Schematic representation of cholinergic neurotransmission system. Acly, ATP-citrate lyase; CoA, coenzyme A; ChAT, choline acetyltransferase; ACh, acetylcholine; VAChT, vesicular acetylcholine transporter; AChE, Acetylcholine esterase; CHT, high affinity choline transporter; AChRs, Acetylcholine receptors. (**B**) ChIP-seq tag profile of the genomic region surrounding a battery of cholinergic genes *ChAT/VAChT*, *CHT*, and *Acly* loci. Each cholinergic gene is indicated, and the blue arrows represent the direction of transcription. Mam cons., mammalian conservation. The ChIP-seq data was deposited in the GEO database (assession no. GSE50993) [20]. (**C**) Schematic representation of the location of the HxRE motifs in each of the 500 bp-long cholinergic gene peaks. The number shows the relative position within the peak (0, the center position of each peak). (**D**) In vivo ChIP assays in dissected E12.5 embryonic spinal cords to monitor the binding of the Isl1-Lhx3-hexamer to the cholinergic enhancers. Schematic representation of the *ChAT* gene is shown on the top. The arrows indicate two sets of primers detecting *ChAT*-enhancer (*ChAT*-enh) and a negative control region lacking the Isl1-Lhx3-binding peak (*ChAT*-neg). Isl1, Lhx3, and NLI were recruited to the cholinergic enhancers in embryonic spinal cords. Error bars indicate standard deviation.

The cholinergic neurotransmission system requires the function of several key factors that are highly expressed in all cholinergic neurons, termed cholinergic pathway genes (Fig. 1A) [4], [5]. Understanding the gene regulatory mechanisms that control the expression of cholinergic pathway genes in different groups of cholinergic neurons will provide crucial insights into the process of cholinergic fate specification in CNS development. Given that each of the cholinergic pathway genes is essential for efficient cholinergic neurotransmission, it is probable that they are up-regulated in a coordinated fashion as neurons acquire cholinergic neuronal identity during vertebrate development. Supporting this possibility, the *vesicular acetylcholine transporter* (*VAChT*, also known as *Slc18a3*) gene is encoded within an intron of the *choline acetyltransferase (ChAT)* gene in all metazoans examined thus far, including *C.elegans*, *Drosophila* and mammals [6]. This unique genomic arrangement suggests that the *ChAT* and *VAChT* genes are co-regulated by a single set of transcription factors. Furthermore, in a subset of cholinergic MNs of *C. elegans*, an Ebf-type transcription factor UNC-3 regulates a battery of cholinergic genes via a shared UNC-3-response motif [7].

Two critical questions remain to be answered. First, is a battery of cholinergic pathway genes coordinately regulated by a common transcription factor in vertebrate CNS, similar to UNC-3-directed control of cholinergic genes in *C.elegans*? Second, could there be a transcription factor(s) that determines cholinergic fate across different types of cholinergic cells in the vertebrate CNS? While very limited information is available for the first question, it is interesting to note, for the latter question, that a LIM homeodomain (LIM-HD) transcription factor Isl1 is expressed in several cholinergic neurons in the spinal cord, hindbrain, forebrain and retina, such as spinal MNs, hindbrain MNs, some FCNs, and starburst amacrine cells [8], [9], [10], [11]. Deletion of *Isl1* gene results in a loss of MNs in the spinal cord and hindbrain [12]. Conditional deletion of *Isl1* gene using a Six3-Cre transgene led to a reduction of restricted FCNs in the brain and cholinergic amacrine cells in the retina [13]. These findings point to the possibility that Isl1 may function as a cholinergic fate determinant in vertebrate CNS. However, it remains unknown whether Isl1 directly control the cholinergic phenotype and, if so, how Isl1 controls the fate of distinct cholinergic cell types whose gene expression patterns and functions are vastly different despite the shared property of cholinergic neurotransmission.

In the developing spinal cord, Isl1 directs motor neuron fate specification by cooperating with another LIM-HD factor Lhx3 [12], [14], [15], [16]. In differentiating MNs, Isl1 binds to Lhx3 and a LIM-interactor NLI (also known as Ldb), thereby forming the Isl1-Lhx3-hexamer complex, also termed MN-hexamer (Fig. S1A) [14], [17]. The combinatorial expression of Lhx3 and Isl1, resulting in the formation of the Isl1-Lhx3-hexamer, is capable of triggering MN specification in chick spinal cord, embryonic stem cells (ESCs), and induced pluripotent stem cells [14], [17], [18], [19], [20]. However, it is unclear whether the Isl1-Lhx3-hexamer directly controls cholinergic neuronal identity, an essential characteristic of MNs. In the developing forebrain, FCNs are derived from the medial ganglionic eminence (MGE) in the ventral telencephalon [21], [22]. A LIM-HD protein Lhx8 is highly expressed in the MGE [21], [23]. The formation of FCNs is severely disrupted in Lhx8-deficient mice [24], [25], [26]. Lhx8 appears to function in combination with Isl1 in driving the differentiation of cholinergic striatal interneurons [27], but the mechanisms by which Lhx8 and/or Isl1 control cholinergic fates in the developing forebrain remain unclear.

In this study, we found that the Isl1-Lhx3-hexamer directly activates the expression of a suite of cholinergic genes by binding to cholinergic gene enhancers that were discovered via ChIP-seq experiments. We also found that Isl1 is co-expressed with Lhx8 and NLI in the embryonic ventral forebrain and forms a hexamer complex with Lhx8 and NLI, named Isl1-Lhx8-hexamer. Interestingly, like the Isl1-Lhx3-hexamer in the spinal cord, the Isl1-Lhx8-hexamer directly controls cholinergic pathway gene expression via the same cholinergic gene enhancer in the forebrain. These findings imply that, despite distinct developmental histories and locations within the nervous system, MNs and some FCNs employ a common molecular mechanism that determines their cholinergic neuronal identity.

## Results

### The Isl1-Lhx3-hexamer is recruited to a battery of cholinergic genes

Given that MNs acquire cholinergic neuronal characteristics as they become specified, we considered the possibility that the Isl1-Lhx3-hexamer, a determinant of the MN fate, regulates expression of a battery of cholinergic genes by directly binding to the enhancer of each cholinergic gene. Intriguingly, our ChIP-seq analysis, which mapped the genomic binding sites of the Isl1-Lhx3-hexamer in mouse embryonic stem cells [20], revealed Isl1-Lhx3-bound peaks in the key cholinergic pathway genes; ChAT, VAChT, high affinity choline transporter (CHT, also known as Slc5a7), a transporter that regulates the uptake of choline from the synaptic cleft into cholinergic neurons, and ATP-citrate lyase (Acly), an enzyme that synthesizes acetyl-CoA (Fig. 1A–C). *ChAT* has a strong peak within an intronic region that lies downstream of the *VAChT* gene, which is itself encoded within the intron of the *ChAT* gene. The *Acly* gene has two strong peaks in intronic regions and one upstream peak, while the *CHT* gene has a peak ∼100 kb downstream of its coding region. All the peaks have at least one hexamer response element (HxRE) (Fig. 1C, Fig. S1B) [20].

To test whether the Isl1-Lhx3-hexamer is recruited to Isl1-Lhx3-bound peak regions of the cholinergic genes in vivo, we purified genomic DNA bound by the Isl1-Lhx3-hexamer from E12.5 embryonic spinal cords using ChIP assays with α-NLI, α-Isl1, and α-Lhx3 antibodies. All three components of the Isl-Lhx3-hexamer bound to the peaks in the cholinergic genes, while they did not bind to the genomic regions without the peaks (Fig. 1D), indicating that the endogenous Isl1-Lhx3-hexamer is recruited to the cholinergic pathway genes in the developing spinal cord. Together, our unbiased, genome-wide ChIP-seq data, along with in vivo ChIP results, strongly suggest that the cholinergic pathway genes are directly activated by the Isl1-Lhx3-hexamer during MN fate specification.

### The Isl1-Lhx3-hexamer upregulates a battery of cholinergic genes in the spinal cord

**Figure 2:**
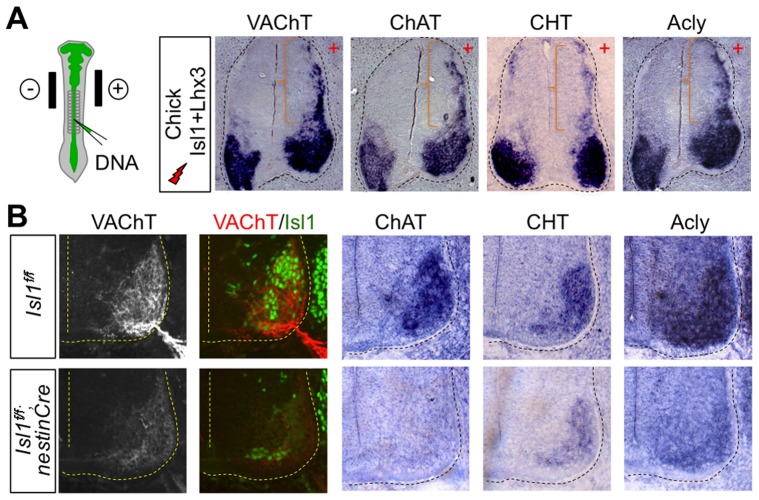
The Isl1-Lhx3-hexamer plays a crucial role in inducing the expression of cholinergic pathway genes in the developing spinal MNs. (**A**) Expression analyses of the cholinergic pathway genes in chick embryos electroporated with Isl1 and Lhx3 using in situ hybridization. The co-electroporation of Isl1 and Lhx3 triggered the ectopic expression of cholinergic genes in the dorsal spinal cord, as marked by brackets. + indicates the electroporated side. (**B**) Expression analyses of the cholinergic pathway genes using either immunohistochemistry or in situ hybridization on the spinal cord of E12.5 *Isl1^f/f^;nestinCre* and littermate control embryos. The ventral quadrant spinal cord is shown. The cholinergic genes are markedly downregulated in *Isl1^f/f^;nestinCre* mice. The remaining VAChT expression is correlated with the residual Isl1 expression in Isl1^f/f^;nestinCre mice, as determined by immunostaining assays.

To test whether the Isl1-Lhx3-hexamer is capable of inducing the expression of multiple cholinergic genes in embryonic spinal cord, we misexpressed Isl1 and/or Lhx3 in the chick neural tube and monitored the expression of cholinergic genes. Co-electroporation of Isl1 and Lhx3 triggered the ectopic expression of a panel of cholinergic genes, including ChAT, VAChT, Acly and CHT, in the dorsal neural tube, while electroporation of Isl1 or Lhx3 alone did not (Fig. 2A, Fig. S2, data not shown). These data indicate that the Isl1-Lhx3-hexamer is capable of upregulating the cholinergic pathway genes in the developing spinal cord.

### Isl1 is required for cholinergic gene expression in the developing spinal cord

To test whether Isl1 is needed for the cholinergic neuronal differentiation in the developing CNS, we deleted the *Isl1* gene in neural progenitors using nestin-Cre [28], [29]. In E12.5 *Isl1^f/f^;nestin-Cre* mice, Isl1 expression in MNs in the ventral spinal cord was greatly reduced (Fig. 2B). In this condition, expression of cholinergic genes, such as Acly, ChAT, VAChT and CHT, is drastically downregulated (Fig. 2B). The weak signal of VAChT was detected only in the remaining Isl1-expressing cells of *Isl1^f/f^;nestin-Cre* mice (Fig. 2B). These results support a role of Isl1 in controlling cholinergic fate decision in the spinal cord.

### Cholinergic enhancers are activated by the Isl1-Lhx3-hexamer

**Figure 3:**
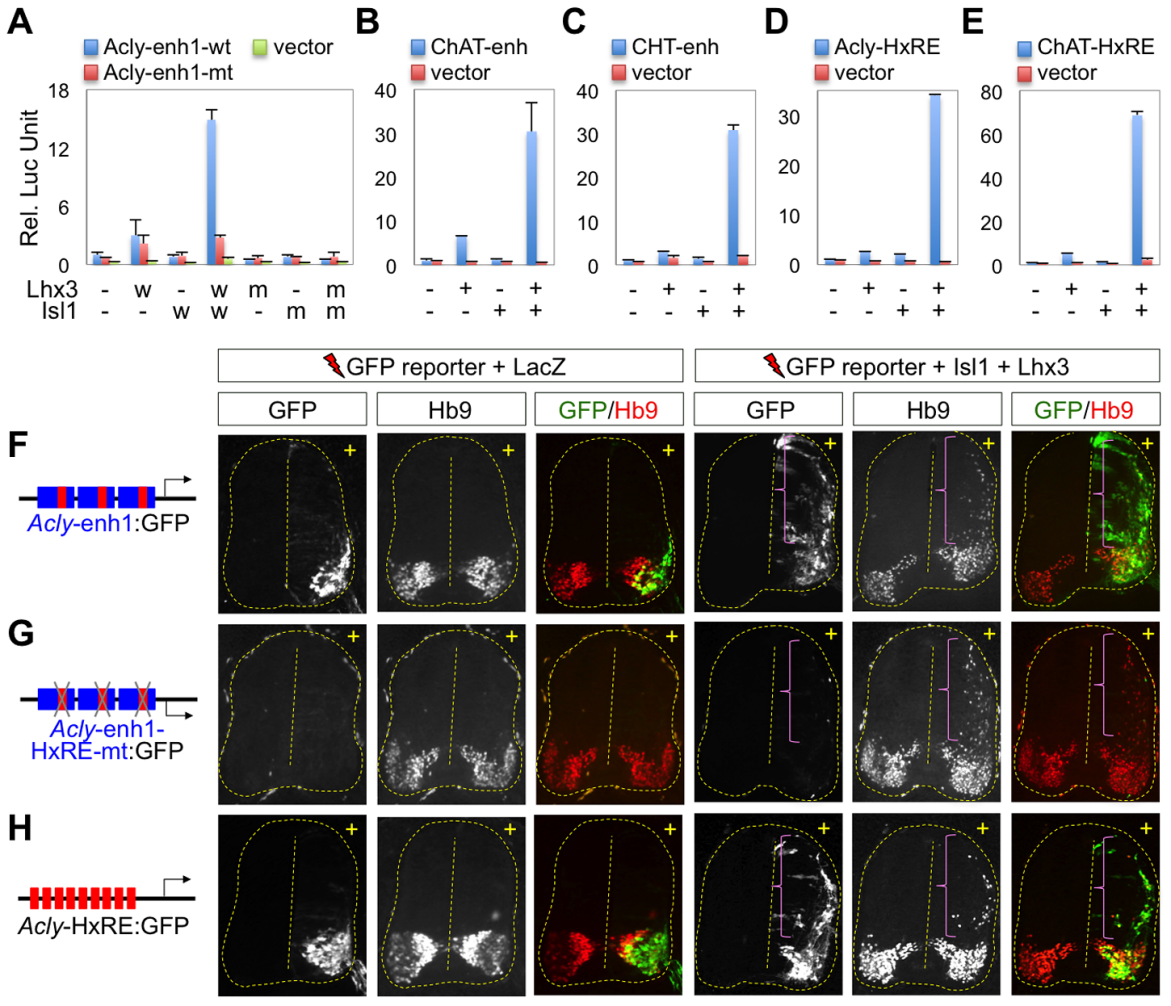
The cholinergic enhancers are activated by the Isl1-Lhx3-hexamer in the developing spinal cord. (**A–E**) Luciferase reporter assays in mouse embryonic P19 cells using *Acly*-enh1-wt:LUC or *Acly*-enh1-mt:LUC, in which HxRE motifs are mutated (A), *ChAT*-enh:LUC (B), *CHT*-enh:LUC (C), *Acly*-HxRE:LUC (D), and *ChAT*-HxRE:LUC (E) reporters with expression vectors as indicated below each graph. The co-expression of Lhx3 wild-type and Isl1 wild-type, indicated as w or +, strongly activated the reporters linked to the cholinergic enhancers, but not the LUC vector alone or *Acly*-enh1-mt:LUC. Lhx3-N211S and Isl1-N230S, the DNA-binding defective missense mutants of Lhx3 or Isl1 that are indicated as m, failed to activate *Acly*-enh1-wt:LUC (A). (A–E) Error bars represent the standard deviation in all graphs. Error bars indicate standard deviation. (**F–H**) GFP reporter activity was monitored in chick embryos electroporated with *Acly*-enh1:GFP (F), *Acly*-enh1-HxRE-mt:GFP (G), and *Acly*-HxRE:GFP (H) reporters with either LacZ or Isl1 plus Lhx3 as indicated above. *Acly*-enh1 and *Acly*-HxRE drove MN-specific GFP expression, and were ectopically activated by co-expression of Isl1 and Lhx3 in the dorsal spinal cord (F, H). *Acly*-enh1-HxRE-mt:GFP did not display GFP expression in MNs and failed to respond to the co-electroporated Isl1 and Lhx3 (G), indicating that the HxRE motif is required for the MN-specific enhancer activity of *Acly*-enh1. + indicates the electroporated side. The areas of ectopic Hb9^+^ MNs, induced by co-expression of Isl1 and Lhx3, are marked by brackets.

To test whether the Isl1-Lhx3-hexamer binding sites in the cholinergic genes act as enhancers to activate the cholinergic pathway genes in the embryonic spinal cord, we first examined whether the Isl1-Lhx3-hexamer activates the transcription of a reporter gene linked to each cholinergic gene peak, referred to here as *ChAT*-enh, *Acly*-enh1 and *CHT*-enh (Fig. 3A–E), using luciferase reporter assays in mouse embryonic P19 cells. As NLI is expressed endogenously in P19 cells, co-expression of exogenous Isl1 and Lhx3 leads to the formation of the Isl1-Lhx3-hexamer [17], [30]. The co-expression of Isl1 and Lhx3 strongly activated the *Acly*:LUC, *ChAT*:LUC and *CHT*:LUC reporters, but not LUC vector alone, in P19 cells, whereas the expression of Isl1 or Lhx3 alone did not (Fig. 3A–C). The *Acly*:LUC reporter with point mutations in the HxRE was not activated by the co-expression of Isl1 and Lhx3 (Fig. 3A). The DNA-binding defective forms of Lhx3 or Isl1 failed to synergize to activate *Acly*:LUC (Fig. 3A), indicating that DNA-binding activity of both Isl1 and Lhx3 is needed to activate the reporter. To further test the role of the HxRE in each enhancer for the potent transcription response to the combination of Isl1 and Lhx3, we generated luciferase reporters that are linked to multiple copies of the HxRE found within *Acly*-enh1, *ChAT*-enh, and *CHT*-enh, respectively. These minimal HxRE reporters were also highly activated by co-expression of Isl1 and Lhx3 (Fig. 3D, E, data not shown), establishing that the HxRE motif mediates activation of the cholinergic enhancers by the Isl1-Lhx3-hexamer. These data establish that the Isl1-Lhx3-hexamer is capable of activating each cholinergic enhancer in the *Acly*, *ChAT/VAChT* and *CHT* genes in heterologous cell types.

To identify in vivo cell types in which the cholinergic enhancers activate gene expression in the developing spinal cord, we electroporated the neural tube of chick embryos with the GFP reporters linked to each cholinergic enhancer in ovo at a time when MNs are being specified. Interestingly, the *Acly*-enh1 drove strong GFP expression in MNs within the developing spinal cord (Fig. 3F, S3A). In contrast, GFP was not expressed in non-MN cell types, despite efficient transfection of those cells following in ovo electroporation (Fig. 3F, Fig. S3A), suggesting that only MNs have the transcriptional machinery that allows activation of *Acly*-enh1. Additionally, *Acly*-enh1 with point mutations in the HxRE motif failed to activate target gene expression in MNs (Fig. 3G, S3B), demonstrating that the HxRE motif is responsible for the MN-specific enhancer activity of the *Acly*-enh1. Furthermore, the multimerized HxRE motifs from the *Acly*-enh1 were sufficient to drive GFP reporter expression in MNs (Fig. 3H, Fig. S3C). Thus, the HxRE motif is necessary and sufficient for the MN-specific enhancer activity of the *Acly*-enh1, suggesting that the endogenous Isl1-Lhx3-hexamer is responsible for *Acly* enhancer activity. Consistent with this idea, co-expression of Isl1 and Lhx3, which assembles the Isl1-Lhx3-hexamer with endogenous NLI and triggers the formation of ectopic MNs in the dorsal spinal cord [14], ectopically activated both *Acly*-enh1:GFP and *Acly*-HxRE:GFP reporters, but it failed to activate *Acly*-enh1 with mutations in HxRE motif (Fig. 3F–H). These data indicate that the Isl1-Lhx3-hexamer is able to activate the *Acly* enhancer in the dorsal neural tube. The expression of Isl1 or Lhx3 alone failed to increase the transcriptional activity of the *Acly*-enh1 or *Acly*-HxRE (Fig. S3D, E). Similarly to the *Acly* enhancer, the *ChAT* enhancer also directed gene expression specifically to MNs and became ectopically activated by the co-expression of Isl1 and Lhx3 (data not shown). Together, these data demonstrate that the endogenous Isl1-Lhx3-hexamer binds to the cholinergic enhancers via the HxRE motifs and triggers the transcription of their target cholinergic genes as MNs are specified in embryonic spinal cords, establishing the Isl1-Lhx3-hexamer as a critical determinant of cholinergic neuronal identity in MNs.

### Isl1 is co-expressed with Lhx8 and NLI in the ventral telencephalon

**Figure 4:**
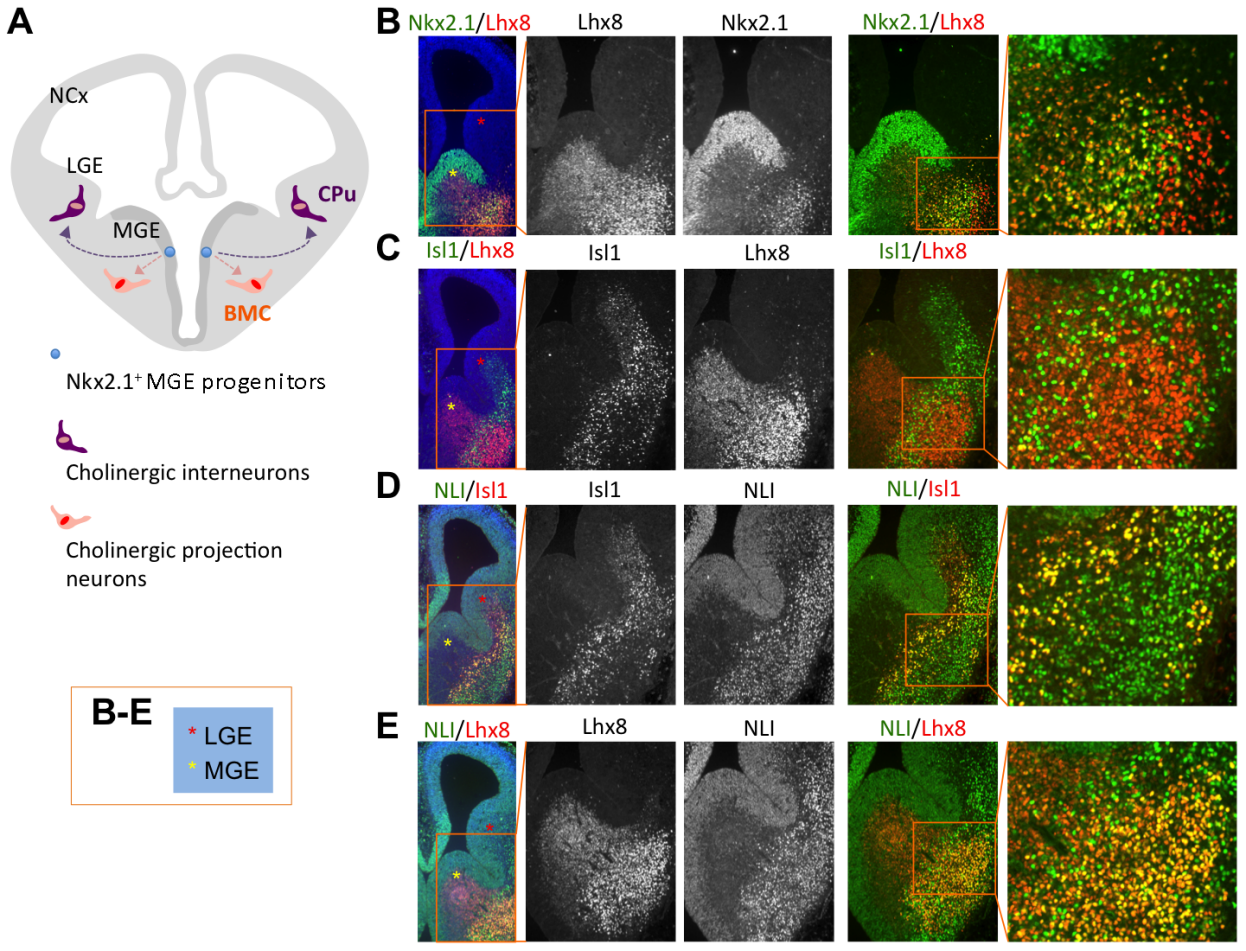
Co-expression of Isl1, Lhx8 and NLI in the developing ventral forebrain. (**A**) Schematic representation of the coronal section of E12.5 forebrain. The MGE produces striatal cholinergic interneurons in the CPu and cholinergic projection neurons in the BMC, which take different migratory paths. NCx, neocortex; MGE, medial ganaglionic eminence; LGE, lateral ganglionic eminence; CPu, Caudate-putamen; BMC, basal meganocellular complex. (**B–E**) Immunohistochemical analyses of expression of Nkx2.1, Isl1, Lhx8, and NLI on coronal sections of E12.5 mouse forebrains. Isl1 is co-expressed with Lhx8 and NLI in the mantle zone of the MGE (yellow asterisk) and LGE (red asterisk).

The coordinated upregulation of cholinergic pathway genes by the Isl1-Lhx3-hexamer in differentiating MNs, along with the previous loss-of-function studies suggesting that Isl1 and Lhx8 play important roles in the generation of FCNs [13], [24], raises the possibility that Isl1 and Lhx8 might form a complex similar to the Isl1-Lhx3-hexamer that drives cholinergic neuronal fate in the developing forebrain. To understand the role of Isl1 and Lhx8 in cholinergic gene expression in the developing forebrain, we determined the expression pattern of Isl1, Lhx3, and and NLI, which might form an Isl1-Lhx3-hexamer-like complex, using double immunohistochemistry analyses. All FCN precursors arise from the Nkx2.1-expressing MGE and preoptic area (POA) in the ventral telencephalon, and take two distinct migratory pathways; tangential migration to form striatal interneurons in the caudate-putamen (CPu) and radial migration to generate projection neurons in the basal forebrain (Fig. 4A) [21], [31]. In E12.5 forebrain, Lhx8 expression is largely confined within the subventricular zone (SVZ) and mantle zone (MZ) of the MGE, but a few Lhx8^+^ cells were found in the MZ of the lateral ganglionic eminence (LGE), which are likely the cells tangentially migrating from the MGE (Fig. 4B, C). In contrast, Isl1 is more abundantly expressed in the SVZ and MZ of the LGE, but is also expressed in the SVZ and MZ of the MGE (Fig. 4C, D). NLI is highly expressed in both MGE and LGE (Fig. 4D, E). Thus, Isl1, Lhx8 and NLI are co-expressed in a substantial fraction of cells in the MGE and LGE at E12.5. A similar expression pattern for Isl1, Lhx8, and NLI was observed in E13.5 forebrain (data not shown).

**Figure 5:**
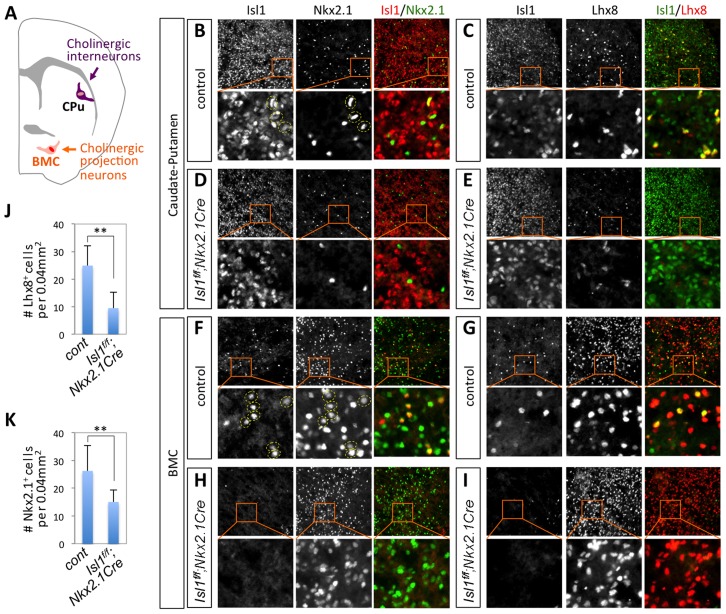
Isl1 is co-expressed with Nkx2.1 and Lhx8 in the CPu and BMC and is important for the formation of Nkx2.1/Lhx8-expressing striatal interneurons. (**A**) Schematic representation of the coronal section of E16.5 forebrain. CPu, Caudate-putamen; BMC, basal meganocellular complex. (**B–I**) Immunohistochemical analyses on the CPu and BMC of E16.5 *Isl1^f/f^;Nkx2.1Cre* and littermate control embryos. Isl1 is co-expressed with Nkx2.1 and Lhx8 in subsets of neurons in the CPu and the BMC (B, C, F, G). The dotted circles depict Isl1/Nkx2.1-double positive cells (D, F). In *Isl1^f/f^;Nkx2.1Cre* embryos, the number of Nkx2.1 or Lhx8-expressing interneurons in the CPu is reduced (B–E), and Isl1^+^ cells in the BMC drastically decreased (F–I). (**J, K**) Quantification of the number of Lhx8- and Nkx2.1-expressing cells in the CPu of E16.5 control and *Isl1* mutant embryos. Histogram shows average ± standard deviation. ** *p*<0.0005 in Student's t-test.

**Figure 6:**
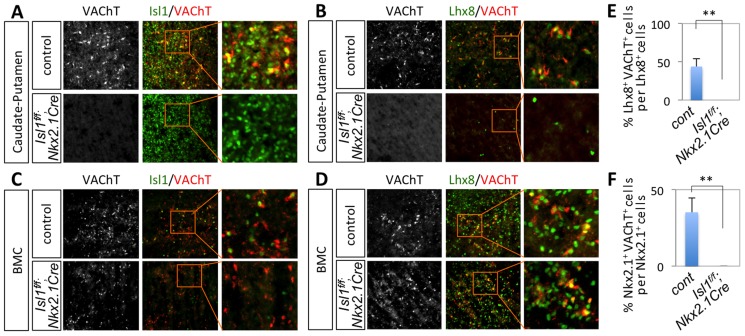
Isl1 is required for the formation of striatal cholinergic interneurons in the developing forebrain. Immunohistochemical analyses on the CPu (A, B) and BMC (C, D) of E16.5 *Isl1^f/f^;Nkx2.1Cre* and littermate control embryos. VAChT^+^ cholinergic interneurons in the CPu failed to form in the MGE-specific *Isl1*-null embryos. (E, F) Quantification of the number of Lhx8^+^VAChT^+^ (E) or Nkx2.1^+^VAChT^+^ cells in the CPu of E16.5 control and *Isl1* mutant embryos. Histogram shows average ± standard deviation. ** *p*<0.0005 in Student's t-test.

By E16.5, VAChT^+^ cholinergic neurons were readily detectable in the CPu and basal meganocellular complex (BMC) (Fig. 5A, 6). Although a majority of CPu cells are derived from the LGE, Nkx2.1^+^ progenitors in the MGE produce distinct subtypes of striatal interneurons in the CPu, including cholinergic interneurons [21], [31]. Most cells in BMC, where a subset of cholinergic projection neurons is located, are generated from the Nkx2.1^+^ MGE [21], [31]. In E16.5 brains, Isl1^+^ cells were much more abundant in the CPu than in the BMC, whereas Nkx2.1^+^ and Lhx8^+^ cells were more abundant in the BMC than in the CPu (Fig. 5B, C, F, G), correlated with their expression at earlier developmental time points (Fig. 4). Despite this distinct pattern of gross expression, a number of Isl1^+^ cells co-expressed Nkx2.1 and Lhx8 in both the CPu and BMC, as shown by double immunohistochemistry analyses (Fig. 5B, C, F, G). Given that Nkx2.1^+^ and Lhx8^+^ striatal interneurons are originated in the MGE [22], [31], a subset of Isl1^+^ cells in the CPu, which co-express Nkx2.1 and Lhx8, is likely interneurons that are produced from the MGE.

### Specific deletion of Isl1 in the MGE leads to a loss of cholinergic interneurons in the CPu

*Isl1^f/f^;nestin-Cre* mice die soon after E12.5, precluding us from observing cholinergic neuronal differentiation in the forebrain. To understand the role of Isl1 in FCN specification in the forebrain, we generated *Isl1^f/f^;Nkx2.1-Cre* mice, in which *Isl1* gene is deleted in cells derived from the Nkx2.1-expressing MGE [31]. As expected, in E16.5 *Isl1^f/f^;Nkx2.1-Cre* mice, the number of Isl1^+^ cells was greatly reduced in the BMC, but not in the CPu where most of Isl1^+^ cells were derived from LGE and thus did not express *Nkx2.1-Cre* (Fig. 5B–I). In the CPu and BMC of the *Isl1*-conditional mutants, neither Isl1/Nkx2.1-double positive cells nor Isl1/Lhx8-co-expressing cells were found (Fig. 5B–I), indicating that *Isl1* is deleted in cells produced from Nkx2.1^+^ MGE and that Isl1/Lhx8-co-expressing cells in the CPu and BMC are derived from the MGE. Interestingly, in the CPu of *Isl1^f/f^;Nkx2.1-Cre* embryos, Lhx8^+^ and Nkx2.1^+^ interneurons were significantly reduced by ∼62% and ∼43%, respectively (Fig. 5J, K), suggesting that Isl1 is required for specification of a subset of Nkx2.1^+^/Lhx8^+^ striatal interneurons.

To monitor cholinergic neuronal differentiation, we performed immunostaining assays with VAChT antibodies. At E16.5, cholinergic neurons were detected in the CPu and BMC, and co-expressed Isl1 and Lhx8 in both areas (Fig. 6A–D). In E16.5 *Isl1^f/f^;Nkx2.1-Cre* mice, however, cholinergic neurons were almost eliminated in the CPu (Fig. 6A, B). While ∼44% Lhx8^+^ cells and ∼35% Nkx2.1^+^ cells were cholinergic in the CPu of control embryos, almost all of Lhx8^+^ and Nkx2.1^+^ neurons did not express VAChT in the CPu of *Isl1^f/f^;Nkx2.1-Cre* mice (Fig. 6E, F). The number of cholinergic neurons in the BMC area of *Isl1^f/f^;Nkx2.1-Cre* embryos appeared to be reduced compared to that in the littermate controls, but the heterogeneity of cholinergic neurons in the BMC made the quantification very challenging (Fig. 6C, D). The remaining cholinergic neurons in the BMC expressed Lhx8 (Fig. 6D). Similar to E16.5, the number of cholinergic neurons remained markedly decreased in the CPu of E17.5 and P2 mice (Fig. S4).

Together, our data indicate that Isl1 and Lhx8 are co-expressed in at least two different populations of FCNs in the CPu and BMC, and that Isl1 function in the MGE-derived cells is required for the specification of cholinergic interneurons in the CPu during forebrain development.

### Isl1 forms the Isl1-Lhx8-hexamer complex with Lhx8 and NLI

**Figure 7:**
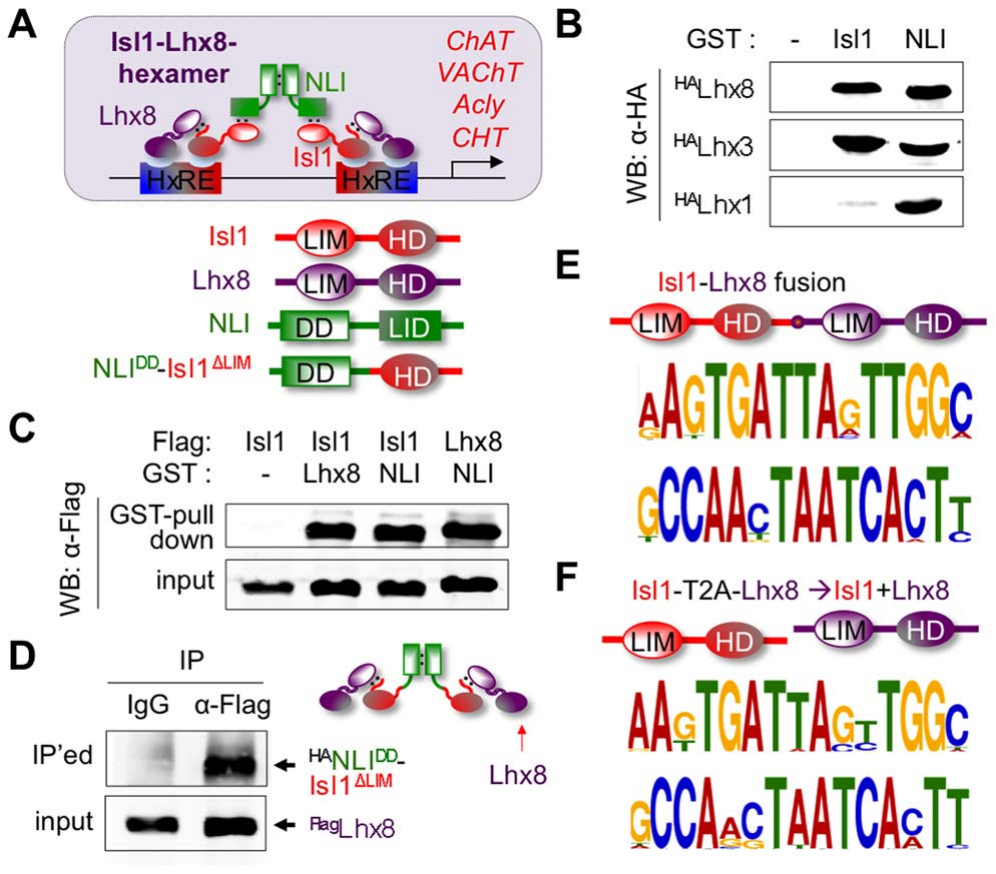
The formation of the Isl1-Lhx8-hexamer complex. (**A**) Schematic representation of the Isl1-Lhx8-hexamer consisting of Isl1, Lhx8 and NLI. The model depicts that the Isl1-Lhx8-complex regulates the cholinergic genes via binding to HxREs. (**B**) In vitro GST-pull down assays. Lhx8 and Lhx3 bind to both Isl1 and NLI with high affinity, whereas Lhx1 binds to only NLI, but not to Isl1. (**C**) GST-pull down assays in HEK293 cells transfected with Flag- and GST-tagged constructs as indicated above. Lhx8 interacts with both Isl1 and NLI in cells. (**D**) CoIP assays in HEK293 cells transfected with Flag-Lhx8 and HA-tagged NLI^DD^-Isl1^ΔLIM^. Lhx8 interact with NLI^DD^-Isl1^ΔLIM^, forming the FCN-hexamer-mimicking complex. (**E, F**) The SELEX methods revealed the high affinity binding sites for Isl1-Lhx8 fusion (E-value, 2.5e-79) and the mixture of Isl1 and Lhx8 (E-value, 2.8e-65). The bottom sequence logo shows reverse complementary sequences of the upper logo.

The co-expression of Isl1 and Lhx8 in FCN precursors and FCNs and requirement of Isl1 and Lhx8 for the specification of a subset of FCNs (Fig. 5, 6, S4) [13], [24] support the possibility that Isl1 and Lhx8 cooperate for the FCN specification by forming a hexamer complex (Fig. 7A), similar to the Isl1-Lhx3-hexamer (Fig. S1). The Isl1-Lhx3-hexamer assembly is dependent on the ability of Lhx3 to interact with Isl1 [14]. Another LIM-HD factor Lhx1 interacts with NLI, a common cofactor of the LIM-HD transcription factors, but does not bind to Isl1, thus forming only a typical LIM tetramer complex consisting of 2NLI:2Lhx1 [14]. Thus, we investigated whether Lhx8 can interact with Isl1, like Lhx3, using in vitro GST-pull down assays (Fig. 7B). As previously shown, Lhx3 interacted with Isl1 as well as NLI, whereas Lhx1 bound only to NLI. Interestingly, Lhx8 strongly associated with both Isl1 and NLI in vitro. Lhx8 also interacted with both Isl1 and NLI in HEK293 cells (Fig. 7C). Combined with the notion that NLI strongly self-dimerizes [32], our data supports a model by which Lhx8, Isl1 and NLI can form a hexameric complex consisting of two NLIs, two Isl1s and two Lhx8 molecules (Figure 7A). We refer to this complex as the Isl1-Lhx8-hexamer.

To further test the formation of Isl1-Lhx8-hexamer complex, we examined whether Lhx8 interacts with NLI^DD^-Isl1^ΔLIM^, in which the dimerization domain (DD) of NLI is fused to LIM domains-deleted Isl1 (Figure 7D). As NLI^DD^-Isl1^ΔLIM^ lacks the LIM-interaction domain (LID) of NLI, it cannot bind to LIM-HD factors via typical interaction interfaces between NLI-LID and LIM-domains of LIM-HD factors, which lead to tetramer formation. Co-immunoprecipitation assays revealed that NLI^DD^-Isl1^ΔLIM^ associated with Lhx8 in cells despite the lack of NLI-LID in this fusion (Fig. 7D), further supporting the formation of the Isl1-Lhx8-hexamer in cells. Together, along with the fact that Lhx8, Isl1 and NLI are co-expressed in FCN precursors in the ventral telencephalon, these results suggest that Lhx8, Isl1, and NLI form the Isl1-Lhx8-hexamer complex in the ventral telencephalon during development.

### The Isl1-Lhx8 complex recognizes a specific DNA motif

To investigate whether the Isl1:Lhx8 dimer, the DNA-binding unit of the Isl1-Lhx8-hexamer, recognizes specific DNA sequences, we performed the unbiased screening method SELEX (for systematic evolution of ligands by exponential enrichment) assay with Isl1, Lhx8, or an Isl1-Lhx8 fusion, in which full-length Isl1 and Lhx8 proteins were linked by a flexible short linker (Fig. 7E). Isl1-Lhx8 highly enriched a 15 nucleotide-long Isl1:Lhx8-binding motif after the third round of SELEX reaction, while Isl1 or Lhx8 failed to enrich any specific DNA sequences. The same motif was also isolated by SELEX with the mixture of Isl1 and Lhx8, which were translated from Isl1-T2A-Lhx8 construct in vitro (Fig. 7F), indicating that the Isl1-Lhx8-binding motif is not an artifact caused by use of the Isl1-Lhx8 fusion protein. These data indicate that Isl1:Lhx8 dimer in the Isl1-Lhx8-hexamer has high affinity to the specific DNA motif. Notably, Isl1-Lhx8-binding motif has a resemblance to the previously identified Isl1:Lhx3-site [17], [20], such as TAAT sequences, but also has unique features (Fig. S5).

### The Isl1-Lhx8-hexamer binds to cholinergic enhancers in developing forebrain

**Figure 8:**
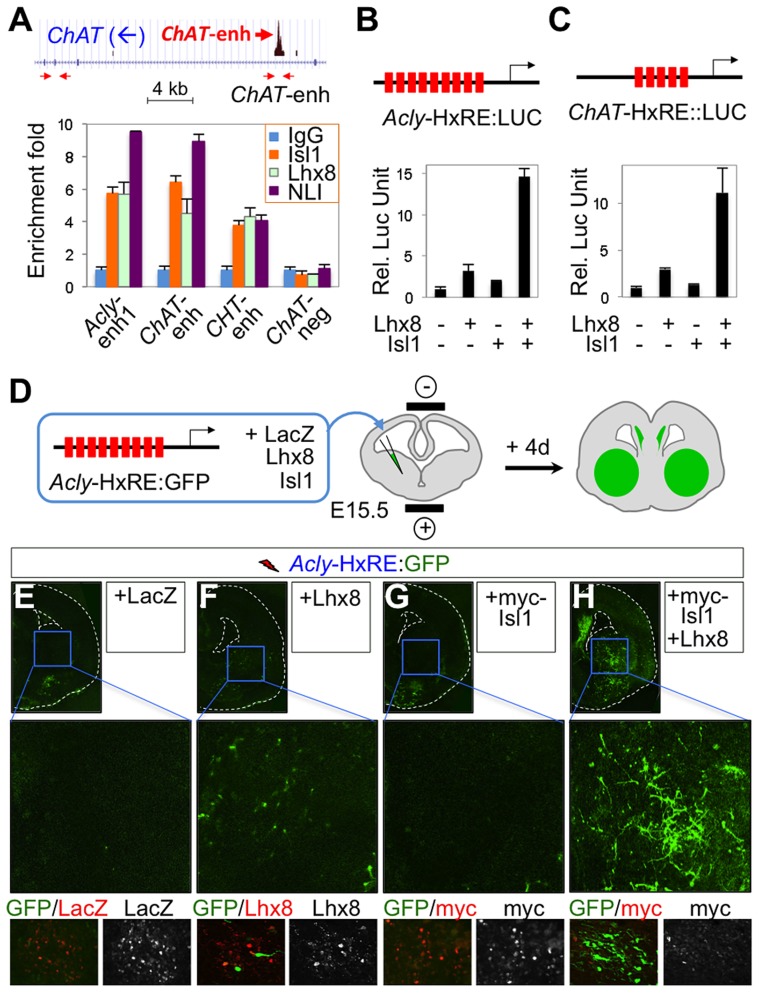
The Isl1-Lhx8-hexamer activates the cholinergic genes. (**A**) ChIP assays with IgG, α-Isl1, α-Lhx8, and α-NLI antibodies in dissected E15.5 embryonic forebrains. The location of two sets of primers in the *ChAT* gene is indicated (arrows). The FCN-hexamer is recruited to the cholinergic enhancers. (**B, C**) Luciferase reporter assays in P19 cells using *Acly*-HxRE:LUC (B), and *ChAT*-HxRE:LUC (C) reporters with vectors indicated below each graph. The co-expression of Lhx8 and Isl1 strongly activated these reporters. Error bars represent the standard deviation in all graphs (A–C). (**D**) Schematic representation of ex vivo electroporation of the ventral forebrain. Sections containing the appropriate regions of the ventral forebrain were focally injected with combinations of plasmids and subjected to slice electroporation, followed by slice culture. The area of transfection was indicated in green. Due to the electroporation process with slices, part of cortex was also transfected with plasmids. (**E–H**) Activation of *Acly*-HxRE:GFP reporter in the ventral forebrains electroporated with constructs, LacZ (E), Lhx8 (F), Isl1 (G), and Isl1 and Lhx8 (H). The co-expression of Isl1 and Lhx8 strongly activated *Acly*-HxRE in the forebrain.

Considering the shared function of Isl-Lhx3-hexamer and Isl1-Lhx8-hexamer in inducing cholinergic genes and the similar features of their binding motifs, it is possible that they bind to the same enhancer regions of cholinergic pathway genes. To test whether, in the developing forebrain, the endogenous FCN-hexamer is recruited to the same cholinergic enhancers identified as targets of the Isl1-Lhx3-hexamer in our ChIP-seq analysis, we performed ChIP assays for the Isl1-Lhx8-hexamer using the dissected E15.5 embryonic forebrains and found that Isl1, Lhx8, and NLI, all components of the Isl1-Lhx8-hexamer, bound to the cholinergic enhancers (Fig. 8A). These results suggest that the cholinergic enhancers recruit the Isl1-Lhx8-hexamer in the embryonic forebrain.

### The Isl1-Lhx8-hexamer activates cholinergic enhancers in the developing forebrain

To test the effect of the Isl1-Lhx8-hexamer on the transcriptional activity of cholinergic enhancers, we performed luciferase reporter assays in P19 cells using the *Acly*-HxRE:LUC and *ChAT*-HxRE:LUC reporter constructs. The co-transfection of Isl1 and Lhx8 activated each cholinergic enhancer, while expression of Isl1 or Lhx8 alone had minimal effect (Fig. 8B, C). These results suggest that the Isl1-Lhx8-hexamer complex triggers the transcriptional activity of cholinergic enhancers.

To investigate the activity of the *Acly* enhancer in the ventral forebrain, we injected the *Acly*-HxRE:GFP reporter along with the expression vectors encoding LacZ, Isl1 or Lhx8 into the ventral regions of E15.5 brain slices. The brain slices were then electroporated, cultured in vitro for four days, and examined for GFP expression (Fig. 8D). Among many LacZ^+^ electroporated cells, only a small number of basal forebrain cells expressed GFP (data not shown). The co-electroporation of Isl1 and Lhx8 along with the *Acly-HxRE*:GFP reporter drastically increased the number of GFP^+^ cells and the levels of GFP expression in the ventral forebrain, whereas expression of Lhx8 or Isl1 alone did not exhibit potent effects on *Acly*-HxRE:GFP. Likewise, the transfection of cortical progenitors using in utero electroporation revealed that the expression of Isl1-Lhx8, but not Isl1 or Lhx8 alone, strongly activates *Acly*-HxRE in the developing cortex (Fig. S6A). These results indicate that the combinatorial expression of Lhx8 and Isl1 promotes *Acly*-HxRE enhancer activity in the developing forebrain.

Together, these data suggest that the Isl1-Lhx8-hexamer is sufficient to activate the cholinergic enhancers in heterologous cells and the developing forebrain.

### Isl1-Lhx8, but not Isl1-Lhx3, induces cholinergic gene expression in the developing forebrain

**Figure 9:**
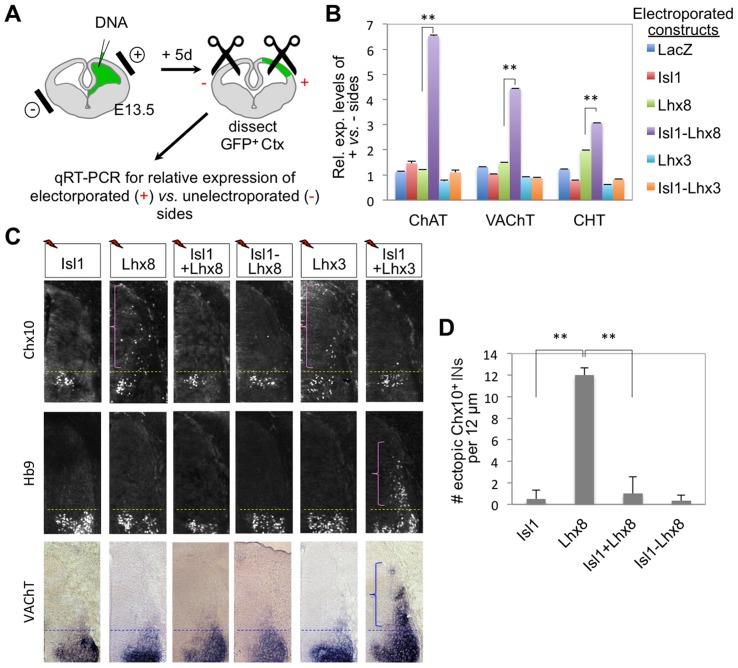
Isl1-Lhx8 induces the expression of cholinergic gene battery in the forebrain, but not in the spinal cord. (**A**) Schematic representation of in utero electroporation of the cortex, followed by quantitative RT-PCR (qRT-PCR) analyses. E13.5 brains were subjected to electroporation after each combination of constructs was injected into the lateral ventricle. The GFP^+^ region of electroporated (+) cortex and the comparable region of unelectroporated (−) cortex were micro-dissected and analyzed for gene expression. (**B**) Expression analyses of the cholinergic pathway genes, *ChAT, VAChT* and *CHT*, in mouse cortices electroporated with constructs, as indicated by color bars. Y-axis indicates the relative expression levels of each cholinergic gene on the electropoated side over the control side. The expression of Isl1-Lhx8 led to upregulation of the cholinergic genes in the cortex, whereas that of Isl1-Lhx3 failed to induce cholinergic genes in this context. Error bars indicate standard deviation. ** *p*<0.0005 in Student's t-test. (**C**) Cell differentiation assays in chick embryos electroporated with constructs as indicated on top. Expression of either Lhx8 or Lhx3 led to the ectopic formation of Chx10^+^ V2 interneurons in the dorsal spinal cord. The electroporation of Isl1 plus Lhx3, but neither Isl1-Lhx8 fusion nor Isl1 plus Lhx8, generated ectopic Hb9^+^VAChT^+^ MNs. Only the electroporated side of the chick spinal cord is shown. Brackets indicate ectopic Chx10^+^ V2 interneurons or Hb9^+^VAChT^+^ MNs, which were formed above the dotted line of endogenous V2 interneurons (Chx10) or MNs (Hb9, VAChT). (**D**) Quantification of ectopic Chx10^+^ V2 interneurons in chick spinal cord upon electroporation of constructs indicated below the graph. Error bars indicate standard deviation. ** *p*<0.0005 in Student's t-test.

The binding and activation of cholinergic enhancers by Isl1-Lhx3 and Isl1-Lhx8 in the spinal cord and forebrain, respectively, prompted us to ask whether both complexes are capable of inducing the cholinergic gene battery irrespective of rostro-caudal positions within the CNS. To address this question, we misexpressed LacZ, Isl1, Lhx8, Lhx3, Isl1-Lhx8 or Isl1-Lhx3, along with EF1 promoter driven-GFP vector to mark the electroporated cells, in the E13.5 mouse cortex using in utero electroporation, and compared the expression levels of cholinergic genes between electroporated and control cerebral hemispheres at E18.5 using quantitative RT-PCR (Fig. 9A). The expression level of transgenes was higher in electroporated sides than in control sides, as expected (Fig. S7A). The expression of Isl1-Lhx8 substantially induced expression of ChAT, VAChT, and CHT in the cortex, compared to expression of Isl1 or Lhx8 alone (Fig. 9B), indicating that Isl1 and Lhx8 function in combination to induce expression of cholinergic genes in the developing forebrain. Interestingly, Isl1-Lhx3 did not trigger cholinergic gene expression in the forebrain (Fig. 9B), despite its potent activity to induce cholinergic pathway genes in the developing spinal cord (Fig. 2A). Moreover, unlike the spinal cord, Isl1-Lhx3 failed to upregulate MN genes, *Isl2*, *Hb9*, and *chodl* [33], in the forebrain (Fig. S7B, data not shown), suggesting that the Isl1-Lhx3-hexamer is unable to turn on the MN gene program in the forebrain. Together, our results strongly support a model whereby the Isl1-Lhx8-hexamer orchestrates upregulation of a battery of cholinergic pathway genes in the developing forebrain.

### The cellular context is critical for the Isl1-Lhx3-hexamer and the Isl1-Lhx8-hexamer to upregulate their target genes

Our results that Isl1-Lhx3 failed to upregulate MN genes and cholinergic genes raise the question of whether Isl1-Lhx8-hexamer is functional in the spinal cord. To address this question, we expressed Lhx8, Isl1, Isl1-Lhx8, or Isl1 plus Lhx8 in chick spinal cord using in ovo electroporation, and monitored cell differentiation and cholinergic gene expression three days post-electroporation. Lhx8 triggered ectopic generation of Chx10^+^ V2a interneurons in the dorsal spinal cord (Fig. 9C, S7C) like Lhx3 [14], underlining the similarity between Lhx8 and Lhx3. Co-expression of Isl1 with Lhx8 blocked Lhx8 from inducing V2a interneurons, suggesting that Isl1 binds to Lhx8 and changes the target gene specificity of Lhx8 as it does with Lhx3 [14] (Fig. 9C, D, S7C). Interestingly, however, co-expression of Isl1 and Lhx8 induced neither ectopic Hb9^+^ MNs nor cholinergic genes in the dorsal spinal cord (Fig. 9C, S7C). Likewise, Isl1-Lhx8 rarely triggered MN formation or cholinergic gene expression in the dorsal spinal cord (Fig. 9C, S7D), indicating that the Isl1-Lhx8-hexamer is ineffective in activating cholinergic gene expression in the spinal cord. Together, our data highlight that the proper cellular context is critical for the Isl1-Lhx3-hexamer and Isl1-Lhx8-hexamer complexes to function in target gene regulation.

### Isl1-Lhx8-hexamer induces cholinergic fates in stem cells

**Figure 10:**
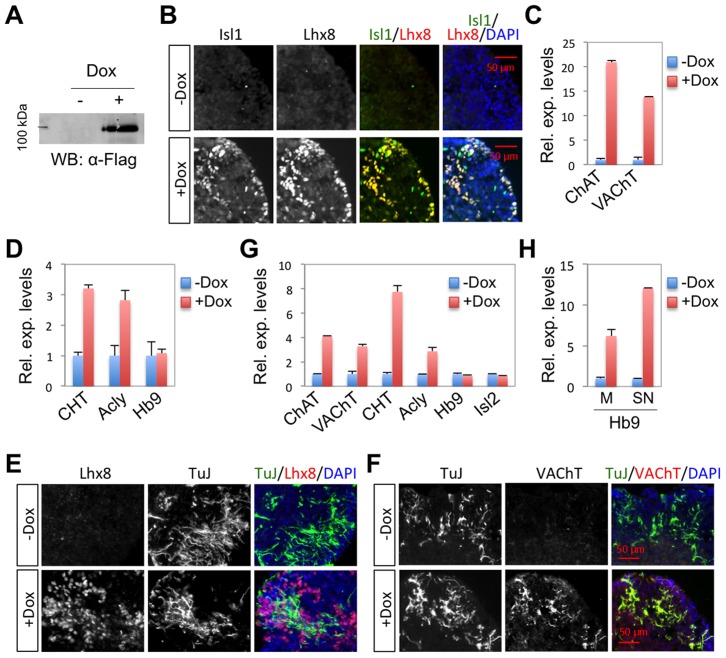
Isl1-Lhx8 induces a cholinergic fate in ESC-derived neurons. (**A, B**) In Isl1-Lhx8-ESCs, the expression of Flag-tagged Isl1-Lhx8 was induced by Dox, as detected by western blotting assays with α-Flag antibodies (A) and immunohistochemistry assays with α-Isl1 and α-Lhx8 antibodies (B). (**C, D**) Quantitative RT-PCR analyses in Isl1-Lhx8-ESCs when cultured as a monolayer. Cholinergic genes, but not the MN gene Hb9, were induced by Isl1-Lhx8. (**E–G**) Cell differentiation analyses in floating EBs derived from Isl1-Lhx8-ESCs, cultured with or without Dox, which triggers expression of Isl1-Lhx8. Immunohistochemical analyses show that Isl-Lhx8 expression induces differentiation of VAChT^+^TuJ^+^ cholinergic neurons (E, F). Quantitative RT-PCR analyses show that cholinergic pathway genes, but not MN genes Hb9 and Isl2, were induced by Isl1-Lhx8 (G). (**H**) Quantitative RT-PCR analyses of Hb9 expression in Isl1-Lhx3-ESCs. Hb9 was induced by Dox treatment, which induces the expression of Isl1-Lhx3 in Isl1-Lhx3-ESCs, when cultured in either monolayer (M) or spinal neuronal differentiation (SN) conditions. Error bars represent the standard deviation in all graphs (C, D, G, H).

Given that the Isl1-Lhx8-hexamer directly regulates the expression of cholinergic gene battery in the developing forebrain, it is possible that the Isl1-Lhx8-hexamer triggers cholinergic neuronal fate in stem cells. To test this possibility, we generated ESCs, in which the expression of Isl1-Lhx8 is induced by doxycycline (Dox), namely Isl1-Lhx8-ESCs (Fig. 10A, B). Isl1-Lhx8 forms the Isl1-Lhx8-hexamer with endogenous NLI in Dox-treated Isl1-Lhx8-ESCs (data not shown). The expression of cholinergic pathway genes, ChAT, VAChT, CHT and Acly, but not a MN gene Hb9, were readily induced by Isl1-Lhx8 under monolayer culture condition (Fig. 10C, D), suggesting that the Isl1-Lhx8-hexamer controls the expression of cholinergic pathway genes in ESCs. We also monitored the cholinergic gene expression in floating culture of embryoid bodies (EBs), which acquire the characteristics of forebrain neural precursors [34]. In the absence of Dox, many TuJ1^+^ neurons were observed in EBs, but VAChT^+^ neurons were hardly detected (Fig. 10E, F). Dox treatment markedly induced VAChT^+^TuJ1^+^ cholinergic neurons in EBs (Fig. 10E, F), suggesting that Isl1-Lhx8 triggers the cholinergic neuronal fate in stem cells. Likewise, RT-PCR also revealed that Isl1-Lhx8 significantly induced the expression of ChAT, VAChT and CHT in EB culture conditions (Fig. 10G). In the same conditions, Isl1-Lhx8 did not induce the expression of MN genes, such as Hb9, Isl2, and Chodl (Fig. 10G, data not shown). In contrast, Isl1-Lhx3 induced Hb9 as well as the cholinergic genes in both monolayer culture and floating embryoid bodies treated with retinoic acid and sonic hedgehog agonist (Fig. 10H, data not shown) [19]. Together, these results indicate that the Isl1-Lhx8-hexamer is capable of triggering the cholinergic neuronal fate, but not MN fate, in stem cells.

## Discussion

**Figure 11:**
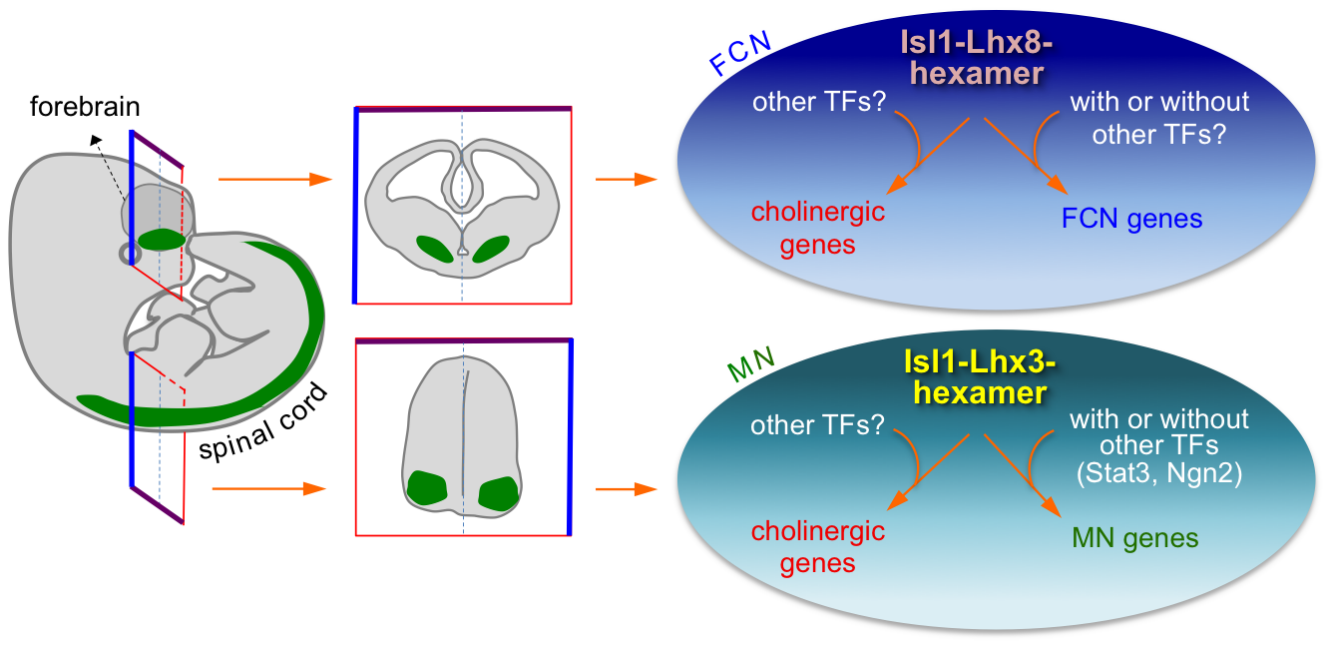
Isl1-Lhx8-hexamer and Isl1-Lhx3-hexamer complexes establish a cholinergic neuronal identity in FCNs and spinal MNs, respectively, by directly upregulating cholinergic gene battery. During CNS development, the cholinergic genes recruit the Isl1-Lhx8-hexamer in the forebrain and the Isl1-Lhx3-hexamer in spinal cord via hexamer-response elements. This recruitment leads to concerted induction of the cholinergic genes, therefore enabling MNs and FCNs to acquire the cholinergic neuronal identity. The Isl1-Lhx8-hexamer and Isl1-Lhx3-hexamer likely induce unique sets of target genes in FCNs and MNs. These hexamers may cooperate with other transcription factors (TFs) in establishing cell type-specific gene expression patterns.

Establishment of correct neurotransmitter characteristics is an essential step of neuronal fate specification, but very little is known about how a battery of genes involved in a specific chemical-driven neurotransmission is coordinately regulated during vertebrate development. In this study, we report that Isl1 directly regulates a battery of genes establishing a cholinergic neurotransmitter characteristic in two developmentally unrelated cell types in vertebrate CNS (Fig. 11). Furthermore, we show that Isl1 does not do this alone, but performs its actions by forming two distinct cell type-specific transcription complexes, the Isl1-Lhx3-hexamer in the spinal cord and the Isl1-Lhx8-hexamer in the forebrain, both of which target common enhancer regions in each of the cholinergic pathway genes.

In *C. elegans*, a set of dopamine pathway genes, which encode dopamine synthesizing enzymes and dopamine transporters, are co-regulated through a specific *cis*-regulatory element that is activated by the ETS transcription factor AST-1 [35]. Likewise, cholinergic pathway genes are co-regulated by a single transcription factor UNC-3 via UNC-3-binding motif in cholinergic MNs of *C.elegans* [7]. Does the vertebrate CNS with much more complex circuits utilize a similar strategy in establishing a particular neurotransmitter identity in multiple types of neurons sharing a neurotransmission system? In vertebrate genome, gene regulatory motifs could occur far away from each gene transcription unit. Thus, identification of a common motif in a battery of neurotransmission-involved genes in vertebrates is much more difficult than in the nematode genome in which the regulatory sequences typically reside in proximity to the transcription start sites. While genome-wide unbiased ChIP-seq approaches could provide a solution to this challenging task, transcription factor(s) controlling a suite of neurotransmission genes need to be identified first to permit ChIP-seq analyses. Expression of Isl1 in multiple cholinergic cell types throughout CNS [8], [9], [10], [11] suggests Isl1 as a good candidate factor to control cholinergic pathway genes. Loss-of-function studies established that Isl1 is required for cholinergic fate specification in spinal MNs, a subset of FCNs, and retinal amacrine cells (this study) [13]. Our study suggests that, to trigger cholinergic neuronal fate, Isl1 functions in combination with other proteins by forming cell type-specific transcription complexes; the Isl1-Lhx3-hexamer in the spinal cord and the Isl1-Lhx8-hexamer in the forebrain. Our ChIP-seq and subsequent analyses revealed that the core set of cholinergic pathway genes shares the binding motif, which recruits the Isl1-Lhx3-hexamer and the Isl1-Lhx8-hexamer in the embryonic spinal cord and forebrain, respectively, and is activated by these complexes. In addition to *ChAT, VAChT, CHT* and *Acly*, our ChIP-seq also uncovered the hexamer-binding peaks in other cholinergic pathway genes, such as *acetylcholine esterase* and a cluster of nicotinic acetylcholine receptors *Chrna5/a3/b4* (data not shown). An important area for future study is whether similar Isl1-containing complexes exist to control cholinergic fate decision in other areas of the CNS, such as retina and hindbrain. Isl1 is co-expressed with Phox2a, a paired-like homeodomain transcription factor, in the cranial MNs of the hindbrain [36]. Interestingly, a recent report shows that Isl1 associates with Phox2a and binds to the same cholinergic enhancer in the *ChAT* gene, which we identified in this study, when co-expressed with Phox2a [37], raising a possibility that Isl1 forms a complex with Phox2a in the hindbrain MNs to control cholinergic gene expression. Together, these results strongly support a model in which the cholinergic pathway genes are concomitantly activated by cell type-specific Isl1-containing complexes during cholinergic neuronal differentiation in the developing CNS (Fig. 11).

While the concept that a defined transcription factor controls the cholinergic gene battery is shared between nematodes and vertebrates, a clear difference is also noteworthy. In *C. elegans* MNs, a single transcription factor UNC-3 serves as a key regulator of the cholinergic pathway genes, whereas, in vertebrate CNS, Isl1-containing cell type-specific transcription complexes control the cholinergic gene battery. The combinatorial utilization of transcription factors is beneficial to generate massively divergent cell types in development. It is possible that regulation of the cholinergic genes by a single transcription factor in ancestral species has been diversified to a transcription complex in vertebrates, as the CNS circuitry becomes more complex. Another possibility is that the hexamer complexes and Ebf transcription factors, vertebrate UNC-3 orthologs, function cooperatively and/or redundantly to control cholinergic genes in the vertebrate CNS. Several findings support a possibility that the Isl1-containing hexamers act together with Ebf. Ebf proteins are expressed in differentiating MNs and ventral forebrain during embryonic development [38], [39], [40]. We found that Ebf1 associates with both types of hexamers in cells (data not shown). Finally, our de novo motif analysis of the Isl1-Lhx3-hexamer-bound ChIP-seq peaks uncovered that the Ebf-binding site is enriched in a subset of the peaks (data not shown). Thus, in the future, it will be interesting to investigate if Ebf factors collaborate with the hexamers in regulating cholinergic genes and other hexamer-targets. Our study demonstrated co-expression of Isl1 and Lhx8 in cholinergic neurons in the embryonic CPu and BMC. Interestingly, while Isl1 is required for cholinergic neuronal differentiation in the CPu, it is dispensable for differentiation of at least a subset of cholinergic neurons in the BMC. Lhx8 alone might be sufficient for the acquisition of the cholinergic phenotype in the remaining FCNs in the BMC. In addition, considering that some cholinergic neurons are still formed in *Lhx8*-deficient mice [24], the Lhx8-independent pathway may be present to trigger cholinergic gene expression in the basal forebrain.

Our finding that both Isl1-Lhx3-hexamer in spinal MNs and Isl1-Lhx8-hexamer in the forebrain bind to the HxRE motif in the cholinergic genes prompts the question of whether these two complexes share other target genes. Given the differences between MNs and FCNs in their functions, synaptic partners, and patterns of cell migration and axon trajectory, it is highly probable that the two complexes have largely separate sets of target genes, which establish MN- or FCN-specific characteristics, while sharing the cholinergic pathway genes as common targets. The Isl1-Lhx3-hexamer and Isl1-Lhx8-hexamer likely bind to similar but distinct sequences, and the HxREs in the cholinergic genes might have characteristics to be recognized by both Isl1-Lhx3-hexamer and Isl1-Lhx8-hexamer. In this respect, it is notable that the most optimal binding motifs for Isl1:Lhx3 or Isl1:Lhx8 identified by the SELEX methods exhibit unique features as well as shared sequences (Fig. S5) [17]. The HxRE motifs in the cholinergic genes show variations from both Isl1:Lhx3- and Isl1:Lhx8-binding sequences (Fig. S1B) [17], [20]. Isl1-Lhx8 failed to bind to the MN-specific enhancer of *Hb9*, which recruits the Isl1-Lhx3-hexamer [30], [41] (data not shown), further suggesting that the Isl1-Lhx8-hexamer and the Isl1-Lhx3-hexamer have unique genomic binding sites. This idea is consistent with the recent finding that the genome occupancy of Isl1 substantially changes depending on whether Isl1 is expressed alone, or co-expressed with Lhx3 or Phox2a, each of which binds Isl1 [37]. Future studies to identify the genome-wide binding sites for the Isl1-Lhx8-hexamer and to compare the target genes and motifs among Isl1-containing cell type-specific complexes will provide important insights into one of fundamental questions of developmental biology; how a single transcription factor directs fates of multiple neuronal types with a common trait. Additional mechanisms likely operate for the Isl1-Lhx3 and Isl1-Lhx8 complexes to choose distinct sets of targets, given that the ability of Isl1-Lhx3-hexamer and Isl1-Lhx8-hexamer to activate target genes is highly dependent on the cellular context. Cholinergic genes were induced only by Isl1-Lhx3 in the spinal cord and only by Isl1-Lhx8 in the forebrain. Moreover, Isl1-Lhx3 readily activated MN genes in the developing spinal cord, but not in the forebrain. First, collaborating transcription factors or cofactors could contribute to the cell context-specific activation of the target genes for each hexamer complex (Fig. 11). The Isl1-Lhx3-hexamer has been shown to cooperate with Neurog2 (Ngn2) and Stat3 in MN gene regulation [20], [30]. It will be interesting to test whether the Isl1-Lhx8-hexamer interacts with other transcription factors, such as Mash1, Olig2, Dbx1/2 or Gbx1/2, to control FCN differentiation in the ventral forebrain. Second, the *in vivo* chromatin context may play a role in cell type-specific gene expression. For instance, MN genes, such as *Hb9* and *Isl2*, may possess transcription-permissive chromatin environment in the spinal cord and transcriptionally inactive chromatin in the forebrain, thus allowing the gene activation by the Isl1-Lhx3-hexamer only in the developing spinal cord, but not in the forebrain. In this regard, it is noteworthy that the activation of Acly-HxRE:GFP reporter gene, which is free from chromain-mediated regulation, is cell context-independent. Both Isl1-Lhx3 and Isl1-Lhx8 was capable of activating the Acly-HxRE:GFP reporter in both the developing spinal cord and forebrain (Fig. 3, S6). Together, our study provides key insights into the gene regulatory logic of cholinergic neuronal differentiation, which would be useful to generate cholinergic neurons for therapeutic or drug screening purposes.

## Materials and Methods

### Ethics statement

All animal procedures were conducted in accordance with the Guidelines for the Care and Use of Laboratory Animals and were approved by the Institutional Animal Care and Use Committee (IACUC) at OHSU.

### DNA constructs

Rat Isl1, Isl1-N230S, and mouse Lhx3, Lhx3-N211S, Lhx8, Lhx1, Isl1-T2A-Lhx3, Isl1-Lhx3 fusion, Isl1-Lhx8 fusion, NLI, and LacZ genes were cloned into pCS2, pcDNA3 (Invitrogen) containing a HA, Flag or myc-epitope tag, or pCIG for expression in mammalian cells and chick embryos and for in vitro transcription and translation reactions. All of these vectors except Lhx8 were previously described [14], [30], [41]. NLI^DD^-Isl1^ΔLIM^ is a fusion of 1-298aa of NLI containing the self-dimerization domain of NLI and 111-349aa of Isl1, which is a C-terminal region of Isl1 that does not include the LIM domains. Isl1-N230S and Lhx3-N211S are missense mutatnts, which are deficient in their ability to directly bind DNA [14]. Isl1, Lhx3, Lhx8, Isl1-Lhx3 and Isl1-Lhx8 were also cloned into the pCIG-2 vector for electroporation of mouse brains. Isl1 and NLI were cloned into the bacterial expression vector pGEX4T-1 (Amersham) for in vitro GST-pull down experiments. Lhx8 and NLI were cloned into the mammalian GST expression vector pEBG for GST-pull down experiments in cell lines. Isl1-Lhx8 was generated by fusing Isl1 full-length and Lhx8 full-length via flexible linker GGSGGSGGSGG. Isl1-T2A-Lhx8 was generated by inserting T2A sequences between full-length Isl1- and full-length Lhx8-coding sequences.

The location of Isl1-Lhx3-bound ChIP-seq peaks for cholinergic genes in mouse genome (mm9) is the following; *ChAT/VAChT*, chr14:33256618–33257117; *CHT*, chr17:54298028–54298480; *Acly*, chr11:100381966–100382465, chr11:100379377–100379876, and chr11:100395147–100395645. Mouse genomic regions covering the *Acly*-enhancer, *ChAT*-enhancer and *CHT*-enhancer were amplified using PCR, and two or three copies of these enhancers were cloned into TK-LUC or synthetic TATA-GFP reporter vectors. Primers to amplify these genomic enhancers are *Acly*-enahncer1, forward 5′- GA AGA TCT TGA TAG CAC ACT ACT TTG CTC TGG, reverse 5′- CG GGA TCC CAG TGA CGC ACG GCG AGC GGG AAG; *ChAT*-enhancer, forward 5′- GA AGA TCT TAC TAA TTG GAT TAA TTG ATT TGC, reverse 5′- CG GGA TCC GGG AAT TAA TAA CTT AGA ATT TGA; *CHT*-enhancer, forward 5′- GA AGA TCT TGA GCA GCC TAT GCC ACA AGG ACA, reverse 5′-CG GGA TCC AGG AAT CCA TCA CAA AGC TAA GAC. AAGCTGATTA sequences in *Acly*-enh1 were mutated to CCGCGCGGCC to generate the *Acly*-enh1-HxRE-mt reporter. *Acly*-HxRE:LUC, *Acly*-HxRE:GFP, *ChAT*-HxRE:LUC and *ChAT*-HxRE:GFP reporters were created by cloning multiple copies of the following duplex oligonucleotides into synthetic TATA-GFP or TK-LUC vectors. *Acly*-HxRE, 5′- CAG AGC TAAT CAG CTTG AGTG GGT-3′; *ChAT*-HxRE 5′- TGG TAC TAAT TGG ATTA ATTG ATT-3′.

### Mice

The generation of *Isl1^f/f^*, *Nestin-Cre*, and *Nkx2.1-Cre* mice has been described previously [28], [29], [31]. *Isl1^f/f^* mice were crossed with *Isl1^f/+^;NesticCre* mice or *Isl1^f/+^;Nkx2.1Cre* mice to generate *Isl1^f/f^;NesticCre* or *Isl1^f/f^;Nkx2.1Cre* embryos, respectively, for analyses. Mouse embryos were collected at the indicated developmental stages, and fixed in 4% paraformaldehyde, embedded in OCT and cryosectioned in 12 µm thickness for immunohistochemistry assays or 18 µm thickness for in situ hybridization with digoxigenin-labeled probes.

### In ovo electroporation

These assays were performed as described [14], [42]. In chick electroporation assays, DNAs were injected into a ∼ Hamburger and Hamilton (HH) stage 13 chick neural tube. The embryos were harvested 3 days post-electroporation and fixed in 4% paraformaldehyde, embedded in OCT and cryosectioned in 12 µm thickness for immunohistochemistry assays or 18 µm thickness for in situ hybridization with digoxigenin-labeled probes. Each set of chick electroporation experiments was repeated independently three to six times with at least three embryos injected with the same combination of plasmids for each experimental set. Representative sets of images from reproducible results were presented.

### Immunohistochemistry and in situ hybridization

For immunohistochemistry assays, the following antibodies were used; rabbit anti-Hb9 [43], mouse anti-Mnr2/Hb9 (5C10, DSHB), rabbit anti-Isl1/2 [9], guinea pig anti-Chx10 [43], rabbit anti-Lhx3 [15], guinea pig anti-VAChT (AB1588, Millipore), goat anti-ChAT (AB144P, Millipore), rabbit anti-GFP (A6455, Molecular Probes), rabbit α-Nkx2.1 (Santa Cruz), guinea pig α-Lhx8 (generated using mouse Lhx8 211–367aa region as antigen), rabbit anti-NLI [44], TuJ1 (Covance) and mouse anti-Flag (Sigma).

For in situ hybridization analyses, cDNA for mouse ChAT, Acly and CHT and chick CHT, Acly, VAChT and ChAT were cloned to pBluescript vector and these vectors were used to generate digoxigenin-labeled riboprobes.

### Co-immunoprecipitation assays and GST-pull down assays

HEK293T cells were seeded onto 10 cm tissue cultures dishes, cultured in DMEM media supplemented with 10% fetal bovine serum, and transfected using Superfect (Qiagen). 48 hours after transfection, cells were harvested and lysed in IP buffer (20 mM Tris-HCl, pH 8.0, 0.5% NP-40, 1 mM EDTA, 150 mM NaCl, 2 mM PMSF, 10% Glycerol, 4 mM Na_3_VO_4_, 200 mM NaF, 20 mM Na-pyroPO_4_, and protease inhibitor cocktail). In these studies, precipitations were performed with either α-Flag antibody (Sigma) or glutathione sepharose beads (GE-Healthcare). The interactions were monitored by western blotting assays using α-Flag (Sigma) and α-HA (Babco) antibodies. Following western blotting with fluorescence-labeled secondary antibodies, the bound fractions of proteins were scanned by the Odyssey imaging system (Li-Cor) following western blotting with fluorescence-labeled secondary antibodies.

In vitro GST-pull down assays were performed as described [45]. BL21 E. coli were transformed with pGEX vector alone, pGEX-Isl1, or pGEX-NLI to express the GST-fusion proteins and lysed by sonication. The GST-fusion proteins were purified by incubating the lysates with glutathione sepharose beads (GE-Healthcare). The beads were then washed and incubated with the putative interacting partners Lhx8, Lhx3 and Lhx1, which were generated in vitro by using the TnT T7 Quick Coupled transcription/translation system (Promega). Bound proteins were eluted by boiling, and were monitored by western blotting assays using α-HA (Babco) antibodies and Odyssey imaging system (Li-Cor).

### SELEX

SELEX was performed as described [46] with proteins in vitro transcribed and translated from the following vectors; Flag- tagged Isl1-Lhx8 fusion, Flag-Isl1, Flag-Lhx8, and Isl1-T2A-Lhx8 which produce both Flag-Isl1 and HA-Lhx8 proteins. The proteins, which were generated by using the TnT T7 Quick Coupled transcription/translation system (Promega), were incubated with a pool of double-stranded oligonucleotides containing a central core region of 22 random nucleotides with identical 5′- and 3′-flanking regions. For each SELEX reaction, ∼30 clones were randomly selected and sequenced. The motif analysis was conducted using Multiple Em for Motif Elicitation (MEME) [47].

### Cell culture and luciferase assays

P19 embryonic carcinoma cells were cultured in α-minimal essential media supplemented with 2.5% fetal bovine serum (FBS) and 7.5% bovine calf serum. For luciferase assays, P19 cells were seeded and incubated for 24 hours, and transient transfections were performed using Lipofectamine 2000 (Invitrogen). An actin promoter-β-galactosidase plasmid was cotransfected for normalization of transfection efficiency, and empty vectors were used to equalize the total amount of transfected DNA. Cells were harvested 36–40 hours after transfection. Cell extracts were assayed for luciferase activity and the values were normalized with β-galactosidase activity. Data are presented as means of triplicate values obtained from representative experiments. All transfections were repeated independently at least four times. Luciferase reporter data are shown in relative activation fold (mean +/− standard deviation).

### Ex vivo embryonic mouse brain electroporation followed by organotypic slice culture and in utero electroporation

The overall procedures for ventral forebrain electroporation and organotypic slice culture were previously described [48]. E15.5 mouse embryos were harvested and brains were dissected and embedded in 3% low melting point agarose dissolved in complete Hanks Balanced Salt solution (cHBSS). 250 µm thick slices of the brains were generated using a Leica VT1200 vibratome. Slices containing the appropriate regions of the ventral forebrain were focally injected with combinations of plasmids. The slices were then mounted on the anode above a 1 mm agarose slice and cHBSS was used to gap the cathode, and electroporated using ECM 830 electroporator (BTX) under the following condition; 60 mV, 5 ms interval pulse, 500 ms delay, and 5 pulses. Immediately after the electroporation, the slices were transferred to transwell inserts (0.4 µm pore size) and cultured for three to five days in vitro with slice media containing 5% heat inactivated horse serum added below the insert at 37°C with 5% CO_2_. Slices were fixed in 4% paraformaldehyde, washed in PBS and analyzed post-fix using immunofluorescence histochemistry.

The overall procedures for ex vivo brain electroporation and organotypic slice culture were previously described [49]. E15.5 mouse embryos were harvested and then the heads were removed and placed in cHBSS. Each combination of DNA constructs mixed with 0.5% Fast Green (Sigma) were injected into the lateral ventricles of isolated E15.5 mouse heads using a Picospritzer III microinjector. The electroporation was carried out on whole heads using ECM 830 electroporator (BTX) under the following condition; 30 mV, 100 ms intervals, 4 pulses, and 100 ms delay. For organotypic slice culture, brains were dissected immediately following electroporation, and embedded in 3% low melting point agarose dissolved in cHBSS. 250 µm thick slices of the brains were generated using a Leica VT1200 vibratome and transferred to transwell inserts (0.4 µm pore size). The slices were then cultured for three to five days in vitro with slice media containing 5% heat inactivated horse serum added below the insert at 37°C with 5% CO_2_. Slices were fixed in 4% paraformaldehyde and analyzed for GFP expression.

Each set of mouse brain electroporation experiments was repeated independently three to six times. For each set of mouse brain electroporation, three to four brain slices were electroporated per condition. Reproducible results were presented in the figures. Confocal images were acquired using a Nikon Eclipse Ti inverted microscope with perfect focus and a motorized stage coupled to a 4 laser line A1 scanning confocal system. Representative sets of images were presented.

For in utero electroporation, timed-pregnant C57BL/6N females were anesthetized at stage E13.5 with isoflurane (4% during induction, 2.5% during surgery), and the uterine horns were exposed by way of laparotomy. 1 µℓ of the expression vector in PBS containing 0.05% fast green (Sigma-Aldrich, St Louis, MO, USA) was injected into the lateral ventricle of the embryo using a 100 mm glass capillary (1B100-4, World Precision Instruments, Inc., USA). Electroporation was performed using Tweezertrodes (diameter, 5 mm; BTX, Holliston, MA, USA) with 5 pulses of 45 V for 50 millisecond duration and 950 millisecond intervals using a square-wave pulse generator (ECM 830; BTX). The uterine horns were then returned to the abdominal cavity, the wall and skin were sutured, and embryos were allowed to continue their normal development and collected for the further analyses at indicated stages.

### RNA extraction and RT-PCR analysis

Total RNAs were extracted using the Trizol (Invitrogen) and reverse-transcribed using the SuperScript III First-Strand Synthesis System (Invitrogen). For quantitative PCR of ChAT, VAChT, Acly and CHT, the following probes and primers predesigned by the TaqMan Gene Expression Assay (Applied Biosystems) for each gene were used with TaqMan Universial Master MixII and 7500 ABI qPCR machine (Applied Biosystems); ChAT (Assay ID-Mm01221882_m1), VAChT (Assay ID-Mm00491465_s1), Acly (Assay ID-Mm01302282_m1), CHT (Assay ID- Mm00452075_m1) and Eukaryotic 18S rRNA Endogenous Control (FAM Dye/MGB Probe, Non-Primer Limited). In addition, the following primers were used with the SYBR green kit (11762-500, Invitrogen) and Mx3000P (Stratagene). *Hb9*, 5′-GTT GGA GCT GGA ACA CCA GT, 5′-CTT TTT GCT GCG TTT CCA TT; ACLY, 5′-GAA GCT GAC CTT GCT GAA CC, 5′-CTG CCT CCA ATG ATG AGG AT; *ChAT*, 5′-CCT GCC AGT CAA CTC TAG CC, 5′-GGA AGC CTT TAT GAT GAG AA; CHT, 5′-GTG GTC TAG CTT GGG CTC AG, 5′-GGC AAT GAG TGC AGA GAC AA; *VAChT*, 5′-TTG ATC GCA TGA GCT ACG AC, 5′-CCA CTA GGC TTC CAA AGC TG; *Hb9*, 5′-GTT GGA GCT GGA ACA CCA GT, 5′-CTT TTT GCT GCG TTT CCA TT; *Isl2*, 5′-GCA AAC TCG CTG AGT GCT TTC, 5′-ACC ATA CTG TTG GGG GTG TC; *Chodl*, 5′-CAG TGG AAT GAC GAC AGG TG, 5′-GGT TCC CAA AGC AAC CAG TA; Isl1, 5′-GAC ATG ATG GTG GTT TAC AGG C, 5′- GCT GTT GGG TGT ATC TGG GAG; Lhx3, 5′-AGA GCG CCT ACA ACA CTT CG, 5′-GGC CAG CGT CTT TCT TCA GT; Lhx8, 5′-CAG TTC GCT CAG GAC AAC AA, 5′-AGC CAT TTC TTC CAA CAT GG; GAPDH, 5′-ACC ACA GTC CAT GCC ATC AC, 5′-TCC ACC ACC CTG TTG CTG TA; and *Cyclophilin A*, 5′-GTC TCC TTC GAG CTG TTT GC, 5′-GAT GCC AGG ACC TGT ATG CT. RT-PCR experiments were performed with three or four independent sets of samples. Data are represented as the mean of duplicate or triplicate values obtained from representative experiments. Error bars represent standard deviation.

### Chromatin immunoprecipitation (ChIP) assays

The ChIP-seq data used for the analysis in this paper has been deposited in the Gene Expression Omnibus (GEO) database (assession no. GSE50993) [20].

To perform the ChIP assays with mouse embryonic tissues, we dissected E12.5 spinal cords or E15.5 forebrains. The microdissected spinal cords from five E12.5 embryos or the forebrains of three E15.5 embryos were combined together for each ChIP reaction with a specific antibody. The tissues were dissociated completely, fixed by 1% formaldehyde for 10 min at room temperature, and quenched by 125 mM glycine. Next, cells were washed with Buffer I (0.25% Triton X-100, 10 mM EDTA, 0.5 mM EGTA, 10 mM Hepes, pH 6.5) and Buffer II (200 mM NaCl, 1 mM EDTA, 0.5 mM EGTA, 10 mM Hepes, pH 6.5) sequentially. Then, cells were lysed with lysis buffer (0.5% SDS, 5 mM EDTA, 50 mM Tris-HCl, pH 8.0, Protease inhibitor cocktail) and were subjected to sonication for DNA shearing. Next, cell lysates were diluted 1∶10 in ChIP buffer (0.5% Triton X-100, 2 mM EDTA, 100 mM NaCl, 50 mM Tris-HCl, pH 8.0, Protease inhibitor cocktail) and, for immunoclearing, were incubated with IgG and protein A agarose beads for one hour at 4°C. Supernatant was collected after quick spin and incubated with appropriate antibodies and protein A agarose beads to precipitate the hexamer/chromatin complex for overnight at 4°C. After pull-down of the hexamer/chromatin complex/antibody complex with protein A agarose beads, the beads were washed with TSE I (0.1% SDS, 1% Triton X-100, 2 mM EDTA, 20 mM Tris-HCl, pH 8.0, 150 mM NaCl), TSE II (same components as in TSE I except 500 mM NaCl) and Buffer III (0.25M LiCl, 1% NP-40, 1% deoxycholate, 1 mM EDTA, 10 mM Tris-HCl, pH 8.0) sequentially for 10 minutes at each step. Then the beads were washed with TE buffer three times. The hexamer/chromatin complexes were eluted in elution buffer (1% SDS, 1 mM EDTA, 0.1M NaHCO_3_, 50 mM Tris-HCl, pH 8.0) and decross-linked by incubating at 65°C overnight. Eluate was incubated at 50°C for more than two hours with Proteinase K. Next, DNA was purified with Phenol/chloroform and DNA pellet was precipitated by ethanol and resolved in water. The purified final DNA samples were subjected to quantitative PCR reactions using the SYBR green kit (11762-500, Invitrogen) and Mx3000P (Stratagene). The total input was used for normalization. All ChIP experiments were repeated independently at least three times. Data are represented as the mean of duplicate or triplicate values obtained from representative experiments, and error bars represent standard deviation.

The following primers were used for ChIP-PCR.

*ChAT*-enhancer

forward 5′-TAC TAA TTG GAT TAA TTG ATT TGC

reverse 5′-GGG AAT TAA TAA CTT AGA ATT TGA

*ChAT*-negative

forward 5′- CTG TGG CTC ATA ACG CTC ATT TTG

reverse 5′- AGT TTG TGG TGG GCC GAG ATG GCA

*Acly*-enh1

forward 5′- TGA TAG CAC ACT ACT TTG CTC TGG

reverse 5′-CAG TGA CGC ACG GCG AGC GGG AAG

*CHT*-enhancer

forward 5′-TGA GCA GCC TAT GCC ACA AGG ACA

reverse 5′- CAT TAG GAG AGC TTG TTC CAG TGA

The following antibodies were used for ChIP-PCR; mouse/rabbit IgG (Santa Cruz), rabbit anti-Isl1 [9], rabbit anti-Lhx3 [15], rabbit anti-NLI [44], and goat anti-Lhx8 (sc-22216, Santa Cruz).

### Generation of Dox-inducible embryonic stem cells (ESCs) and differentiation of ESCs

The generation of Isl1-Lhx3-ESCs was described previously [19]. To generate Isl1-Lhx8-ESCs, the A172LoxP ES cell line [50] was maintained in an undifferentiated state on 0.1% gelatin-coated dishes in the ESC growth medium that consisted of Knockout DMEM, 10% FBS, 0.1 mM non-essential amino acids, 2 mM L-glutamine, 0.1 mM β-mercaptoethanol and recombinant leukemia inhibitory factor (LIF, 1000 units/ml, Chemicon). Flag-tagged Isl1-Lhx8 fusion was inserted into Tet-inducible plasmid p2Lox. The Isl1-Lhx8 vector was co-transfected with pSALK-Cre into the A172LoxP ES cell line using Lipofectamine 2000 (Invitrogen). Stable transfectants were isolated by selection with neomycin (G418, 400 µg/ml) for seven days. Dox-dependent induction of Flag-Isl1-Lhx8 expression was monitored by western blotting and immunohistochemical analyses using α-Isl1, α-Lhx8 and α-Flag antibodies.

To induce cell differentiation, Embryoid bodies (EBs) were formed and cultured for 2 days using the hanging drop method (1×10^3^ ESCs per 20 µℓ drop). Hanging drops were transferred to suspension culture in 6 well low attachment dishes and cultured. EBs were cultured without or with doxycycline (2 µg/ml) for 2–5 days in the ESC medium without LIF or in the differentiation medium that contains KnockOut serum replacement (Life technologies). Then, EBs were collected for either RT-PCR or immunohistochemical analyses.

## Supporting Information

Figure S1 The binding sites of the Isl1-Lhx3-hexamer in the cholinergic pathway genes. (**A**) Schematic representation of the Isl1-Lhx3-hexamer composed of two Isl1, two Lhx3 and two NLI molecules. The Isl1-Lhx3-hexamer binds to HxRE (hexamer-response element) in target MN genes and activates their transcription. (**B**) The location and sequences of the putative HxRE motifs in each of the cholinergic gene peaks. The bars represent 500 bp-long Isl1-Lhx3-bound ChIP-seq peaks associated with cholinergic genes. The number below each bar shows the relative position of the HxRE within each peak (0, the center position of each peak). The core sequences of the HxRE motifs are shown in red.
(TIFF)

Figure S2 The co-expression of Isl1 and Lhx3, but not expression of Isl1 or Lhx3 alone, triggers ectopic expression of cholinergic genes in the dorsal spinal cord. The chick neural tube was electroporated with LacZ, Isl1, Lhx3 or Isl1 plus Lhx3, were analyzed for expression of VAChT or ChAT using in situ hybridization. The efficiency of electroporation was determined by immunostaining with α-LacZ, α-myc, or α-Lhx3 antibodies. + indicates the electroporated side. Brackets mark ectopic induction of cholinergic genes.
(TIFF)

Figure S3 The Isl1-Lhx3-hexamer activates the cholinergic enhancer via HxRE motifs in the developing spinal cord. (**A–C**) GFP reporter activity was monitored in chick embryos electroporated with *Acly*-enh1:GFP (A), *Acly*-enh1-HxRE-mt:GFP (B), and *Acly*-HxRE:GFP (C) reporters with LacZ. LacZ expression marks the electroporated cells. *Acly*-enh1 and *Acly*-HxRE drove MN-specific expression of GFP, while *Acly*-enh1-HxRE-mt failed to do so, indicating that the HxRE motif is required for the MN-specific enhancer activity of *Acly*-enh1. (**D, E**) Co-expression of Isl1 and Lhx3 activated *Acly*-enh1 (D) and *Acly*-HxRE (E) in the dorsal spinal cord as marked by brackets, but Isl1 or Lhx3 alone was not sufficient to activate the reporters in the dorsal spinal cord. + indicates the electroporated side.
(TIFF)

Figure S4 Isl1 is required for the formation of cholinergic interneurons in the CPu of the developing forebrain. Immunohistochemical analyses on the CPu of *Isl1^f/f^;Nkx2.1Cre* and littermate control mice at E17.5 (A) or P2 (B). VAChT^+^ cholinergic neurons in the CPu failed to form in the MGE-specific *Isl1*-null embryos.
(TIFF)

Figure S5 The comparison of HxRE motifs for Isl1-Lhx8 or Isl1-Lhx3 complexes. The Isl1-Lhx8-binding motif was identified by SELEX. The Isl1-Lhx3-binding motifs were identified by SELEX or ChIP-seq assays [17], [20]. ChIP-seq assays uncovered HxRE-long and HxRE-short motifs [20].
(TIFF)

Figure S6 *Acly*-HxRE was activated by both Isl1-Lhx8 and Isl1-Lhx3. (**A**) GFP reporter activity was monitored in mouse cortices electroporated in utero with *Acly*-HxRE:GFP along with various constructs indicated above each image. The *Acly*-HxRE was highly activated by Isl1-Lhx8 or Isl1-Lhx3. (**B**) GFP reporter activity was monitored in chick embryos electroporated with *Acly*-enh1:GFP and Isl1-Lhx8. Expression of Isl1-Lhx8 activated *Acly*-HxRE in the dorsal spinal cord, as marked by brackets, but failed to induce ectopic Hb9^+^ MNs.
(TIFF)

Figure S7 Analyses of mouse or chick embryos electroporated Isl1, Lhx3, Lhx8, Isl1-Lhx3 or Isl1-Lhx8. (**A, B**) Gene expression analyses in mouse cortices electroporated in utero with constructs, as indicated by color bars. In utero electroporation was performed with E13.5 brains and the qRT-PCR analyses were done in E18.5 cortices. Y-axis indicates the relative expression levels of each gene, shown in the x-axis, on the electropoated side over the control side. The expression from the electroporated constructs was detected by qRT-PCR (A). Expression of Isl1 and Lhx3, either alone or in combination, failed to induce MN genes Isl1 and Chodl. The expression levels of Hb9 were below detection level in qRT-PCR analyses in any of these conditions. Error bars indicate standard deviation. (**C, D**) Cell differentiation assays in chick embryos electroporated with constructs as indicated on top. Expression of either Lhx8 or Lhx3 led to the ectopic formation of Chx10^+^ V2 interneurons in the dorsal spinal cord, which was suppressed by co-expression of Isl1. Among all conditions, only co-expression of Isl1 and Lhx3 resulted in ectopic upregulation of Hb9 or VAChT. + indicates the electroporated side. Brackets indicate ectopic Chx10^+^ V2 interneurons or Hb9^+^VAChT^+^ MNs.
(TIFF)
