# Supplementary figures and images for: Isl1 Directly Controls a Cholinergic Neuronal Identity in the Developing Forebrain and Spinal Cord by Forming Cell Type-Specific Complexes
Source: PLoS Genet. 2014 Apr 24;10(4):e1004280. doi: 10.1371/journal.pgen.1004280 (PMC3998908; doi:10.1371/journal.pgen.1004280)

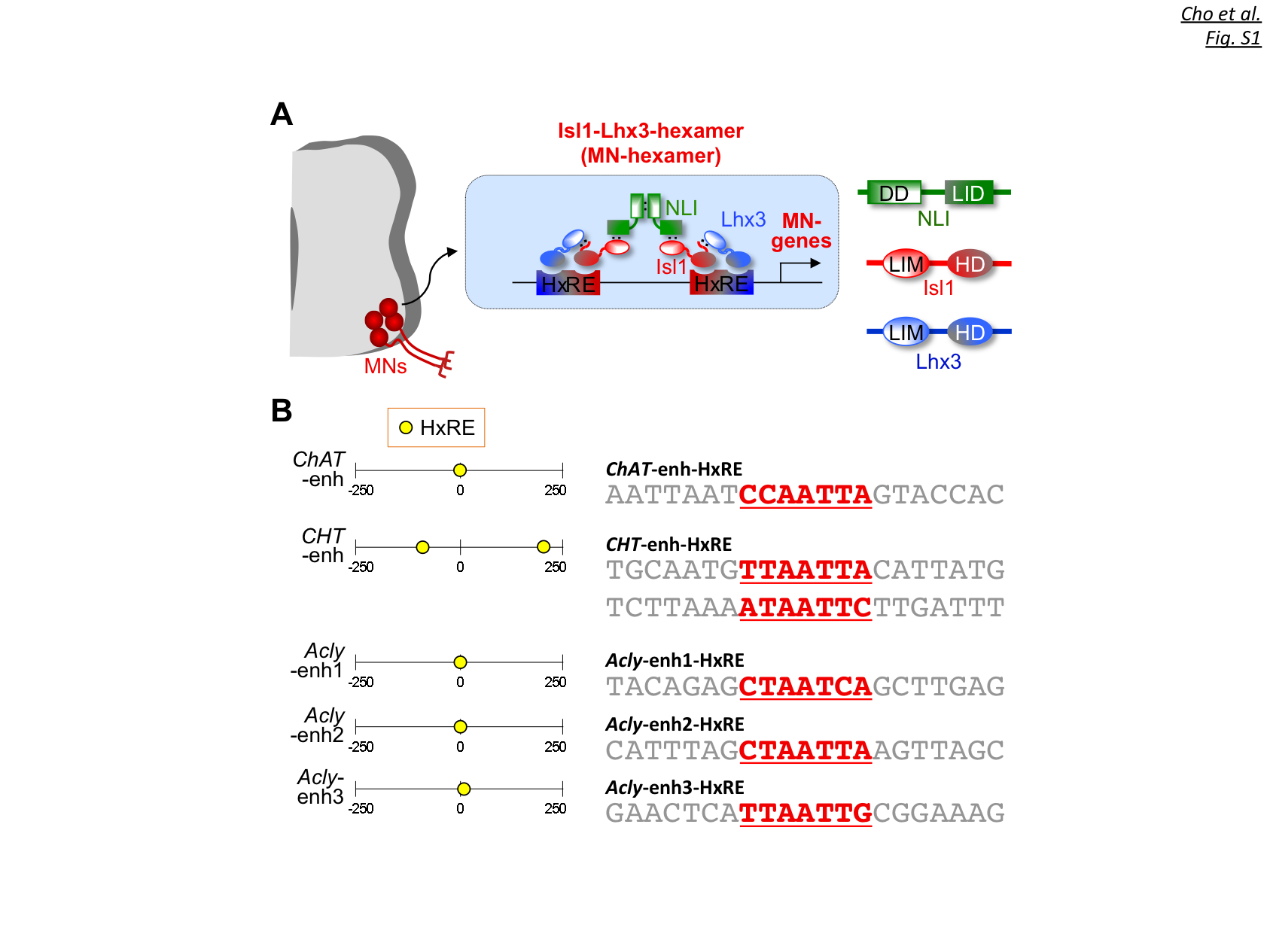

Supplement: Figure S1 — The binding sites of the Isl1-Lhx3-hexamer in the cholinergic pathway genes. (A) Schematic representation of the Isl1-Lhx3-hexamer composed of two Isl1, two Lhx3 and two NLI molecules. The Isl1-Lhx3-hexamer binds to HxRE (hexamer-response element) in target MN genes and activates their transcription. (B) The location and sequences of the putative HxRE motifs in each of the cholinergic gene peaks. The bars represent 500 bp-long Isl1-Lhx3-bound ChIP-seq peaks associated with cholinergic genes. The number below each bar shows the relative position of the HxRE within each peak (0, the center position of each peak). The core sequences of the HxRE motifs are shown in red. (TIFF) [file pgen.1004280.s001.tiff]

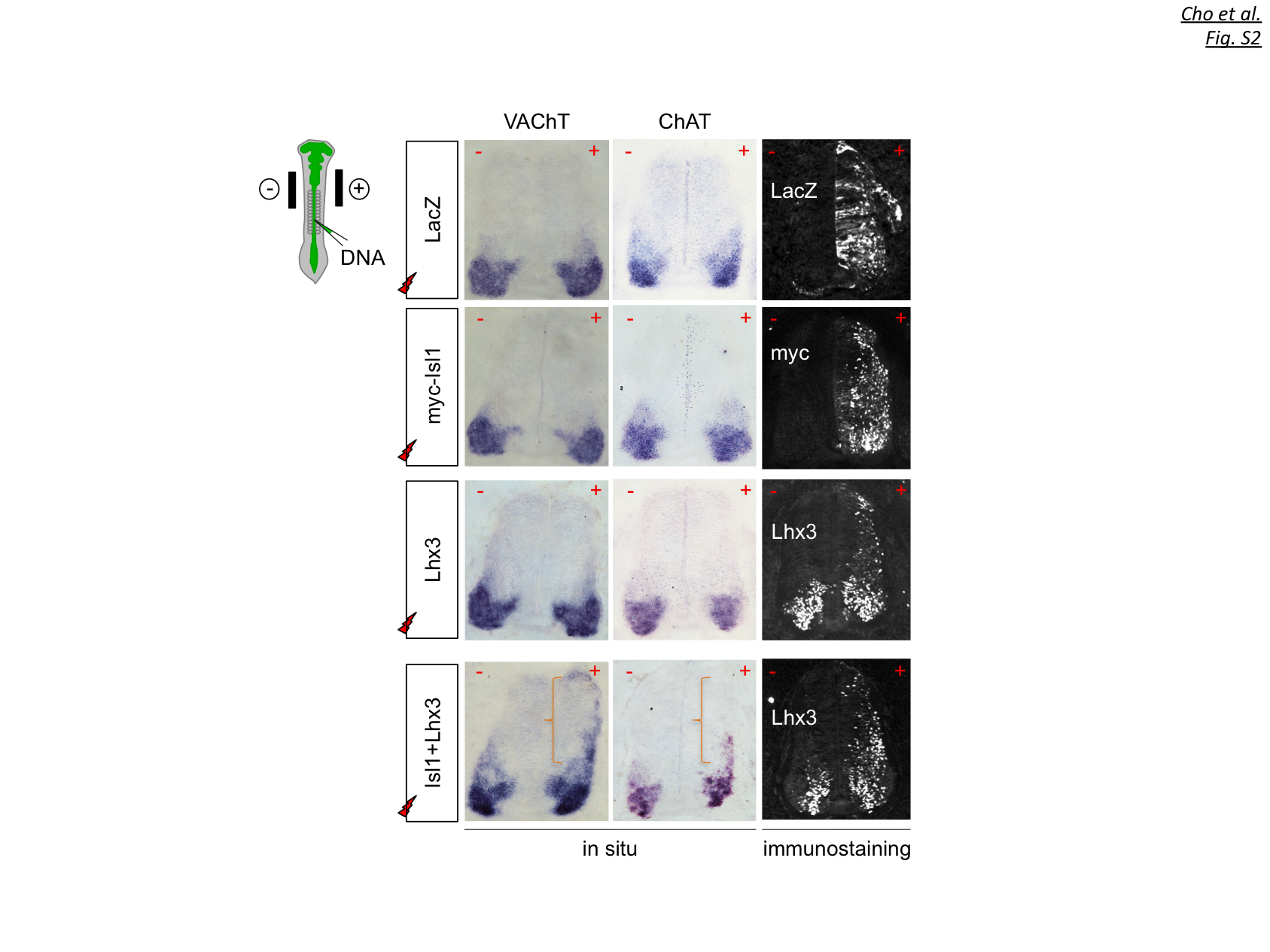

Supplement: Figure S2 — The co-expression of Isl1 and Lhx3, but not expression of Isl1 or Lhx3 alone, triggers ectopic expression of cholinergic genes in the dorsal spinal cord. The chick neural tube was electroporated with LacZ, Isl1, Lhx3 or Isl1 plus Lhx3, were analyzed for expression of VAChT or ChAT using in situ hybridization. The efficiency of electroporation was determined by immunostaining with α-LacZ, α-myc, or α-Lhx3 antibodies. + indicates the electroporated side. Brackets mark ectopic induction of cholinergic genes. (TIFF) [file pgen.1004280.s002.tiff]

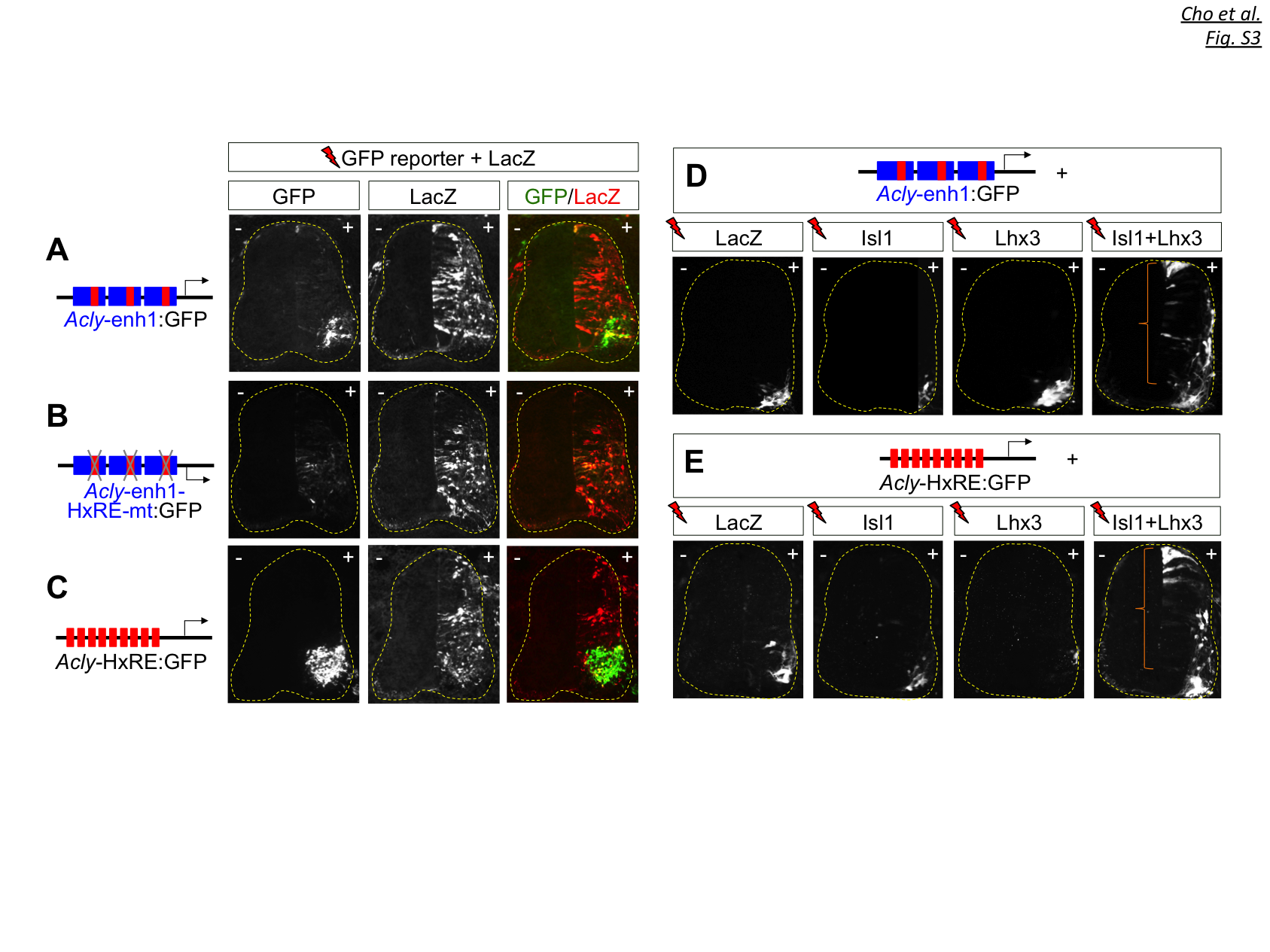

Supplement: Figure S3 — The Isl1-Lhx3-hexamer activates the cholinergic enhancer via HxRE motifs in the developing spinal cord. (A–C) GFP reporter activity was monitored in chick embryos electroporated with Acly-enh1:GFP (A), Acly-enh1-HxRE-mt:GFP (B), and Acly-HxRE:GFP (C) reporters with LacZ. LacZ expression marks the electroporated cells. Acly-enh1 and Acly-HxRE drove MN-specific expression of GFP, while Acly-enh1-HxRE-mt failed to do so, indicating that the HxRE motif is required for the MN-specific enhancer activity of Acly-enh1. (D, E) Co-expression of Isl1 and Lhx3 activated Acly-enh1 (D) and Acly-HxRE (E) in the dorsal spinal cord as marked by brackets, but Isl1 or Lhx3 alone was not sufficient to activate the reporters in the dorsal spinal cord. + indicates the electroporated side. (TIFF) [file pgen.1004280.s003.tiff]

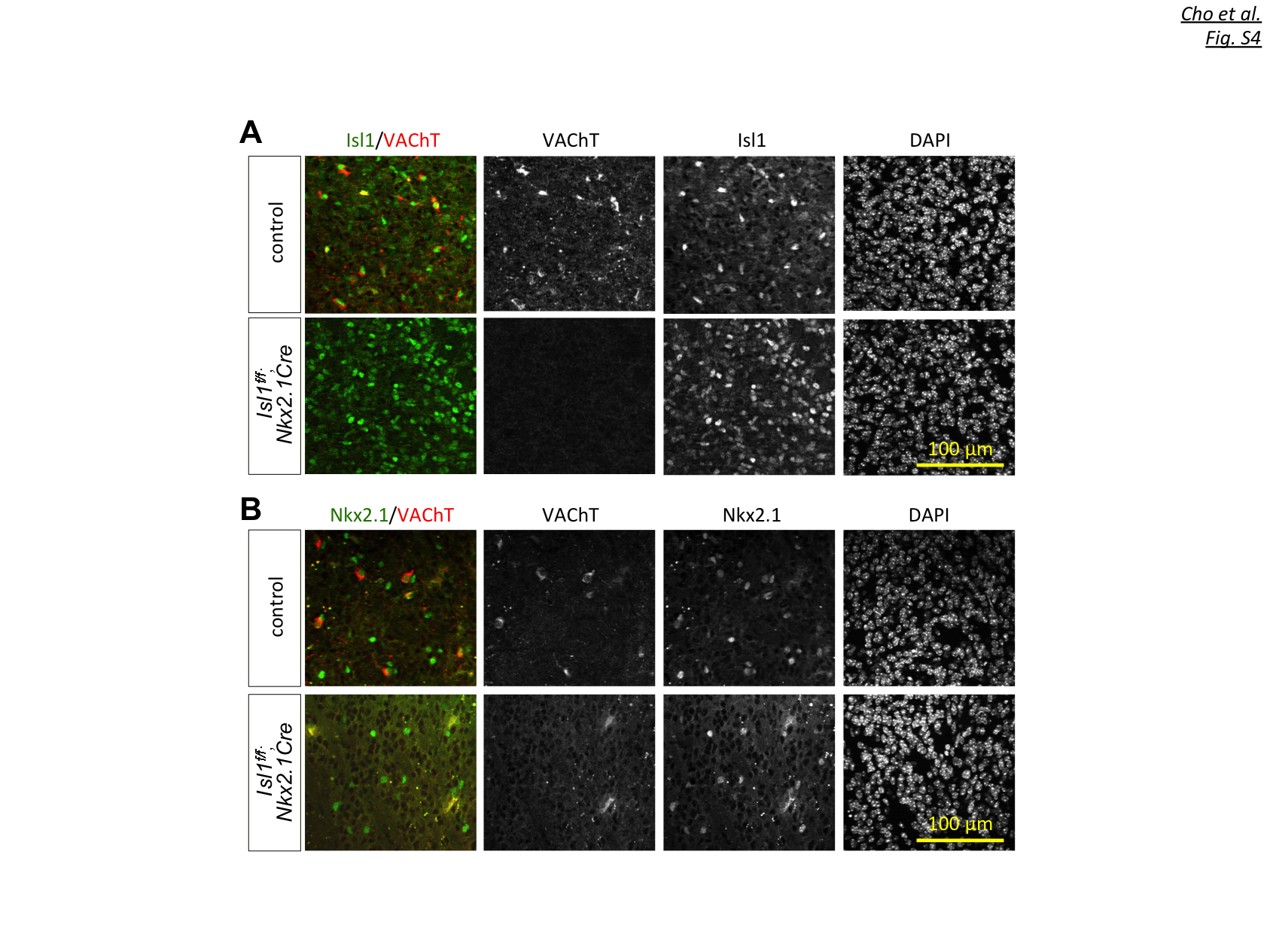

Supplement: Figure S4 — Isl1 is required for the formation of cholinergic interneurons in the CPu of the developing forebrain. Immunohistochemical analyses on the CPu of Isl1f/f;Nkx2.1Cre and littermate control mice at E17.5 (A) or P2 (B). VAChT+ cholinergic neurons in the CPu failed to form in the MGE-specific Isl1-null embryos. (TIFF) [file pgen.1004280.s004.tiff]

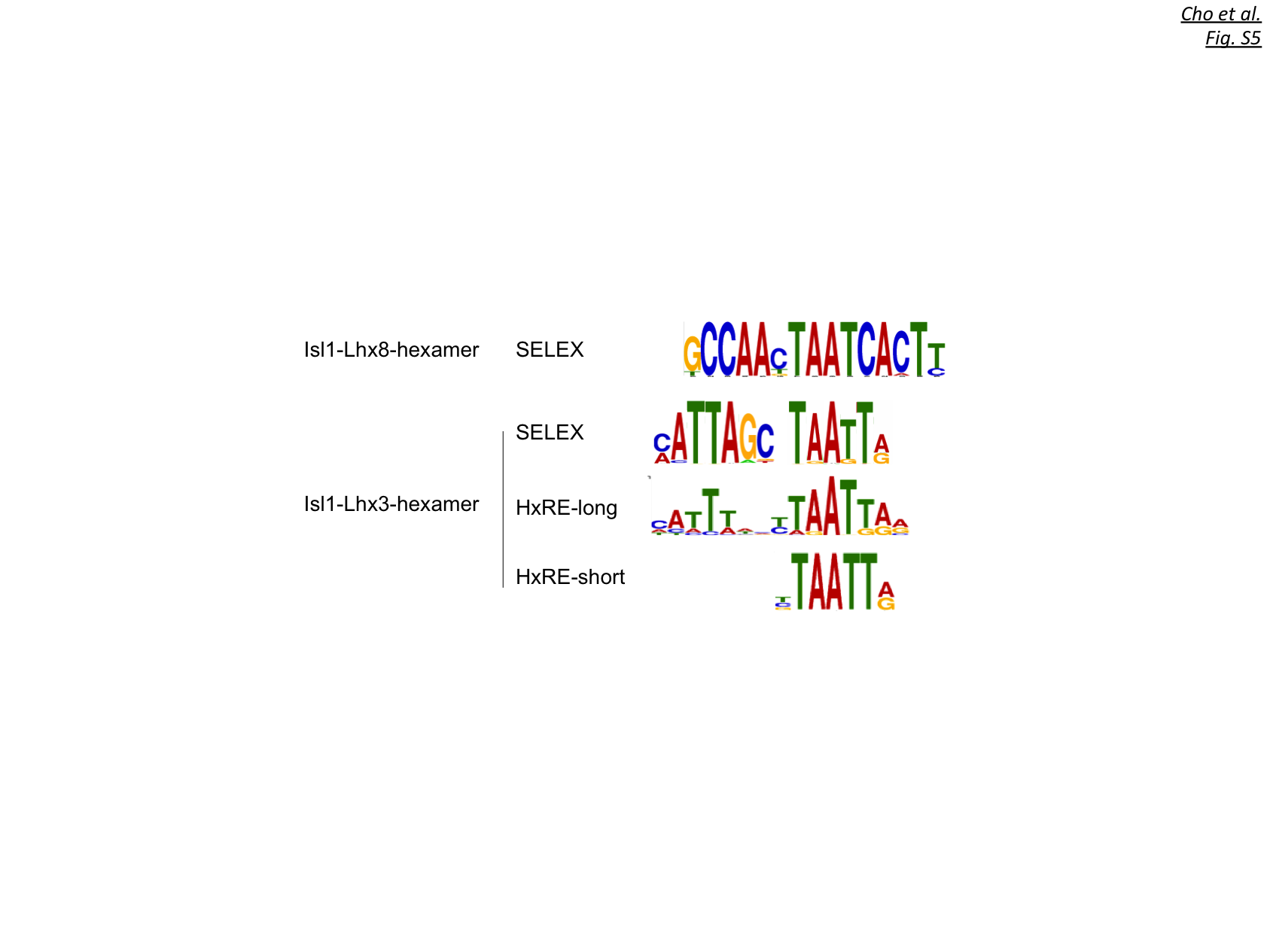

Supplement: Figure S5 — The comparison of HxRE motifs for Isl1-Lhx8 or Isl1-Lhx3 complexes. The Isl1-Lhx8-binding motif was identified by SELEX. The Isl1-Lhx3-binding motifs were identified by SELEX or ChIP-seq assays [17], [20]. ChIP-seq assays uncovered HxRE-long and HxRE-short motifs [20]. (TIFF) [file pgen.1004280.s005.tiff]

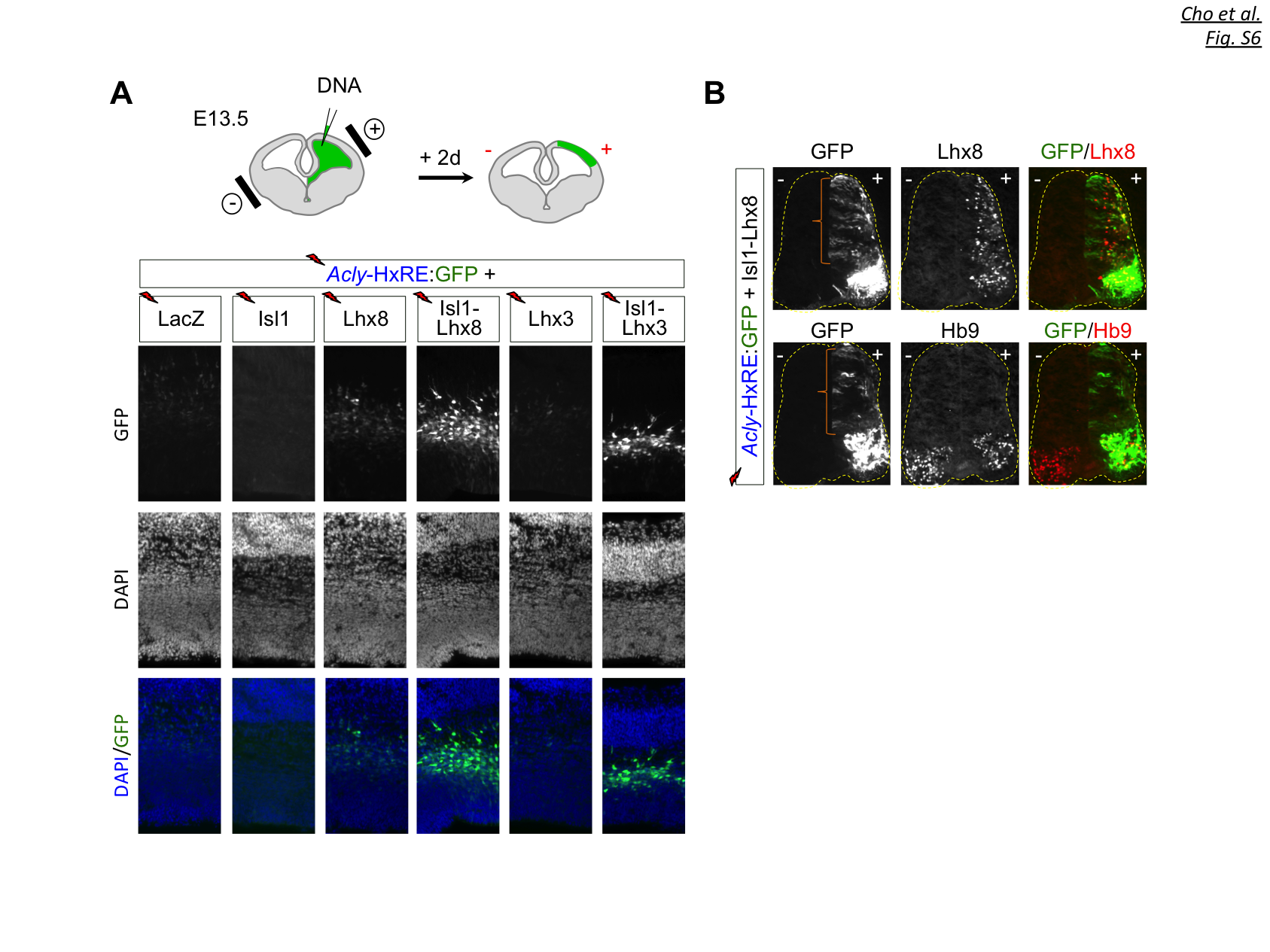

Supplement: Figure S6 — Acly-HxRE was activated by both Isl1-Lhx8 and Isl1-Lhx3. (A) GFP reporter activity was monitored in mouse cortices electroporated in utero with Acly-HxRE:GFP along with various constructs indicated above each image. The Acly-HxRE was highly activated by Isl1-Lhx8 or Isl1-Lhx3. (B) GFP reporter activity was monitored in chick embryos electroporated with Acly-enh1:GFP and Isl1-Lhx8. Expression of Isl1-Lhx8 activated Acly-HxRE in the dorsal spinal cord, as marked by brackets, but failed to induce ectopic Hb9+ MNs. (TIFF) [file pgen.1004280.s006.tiff]

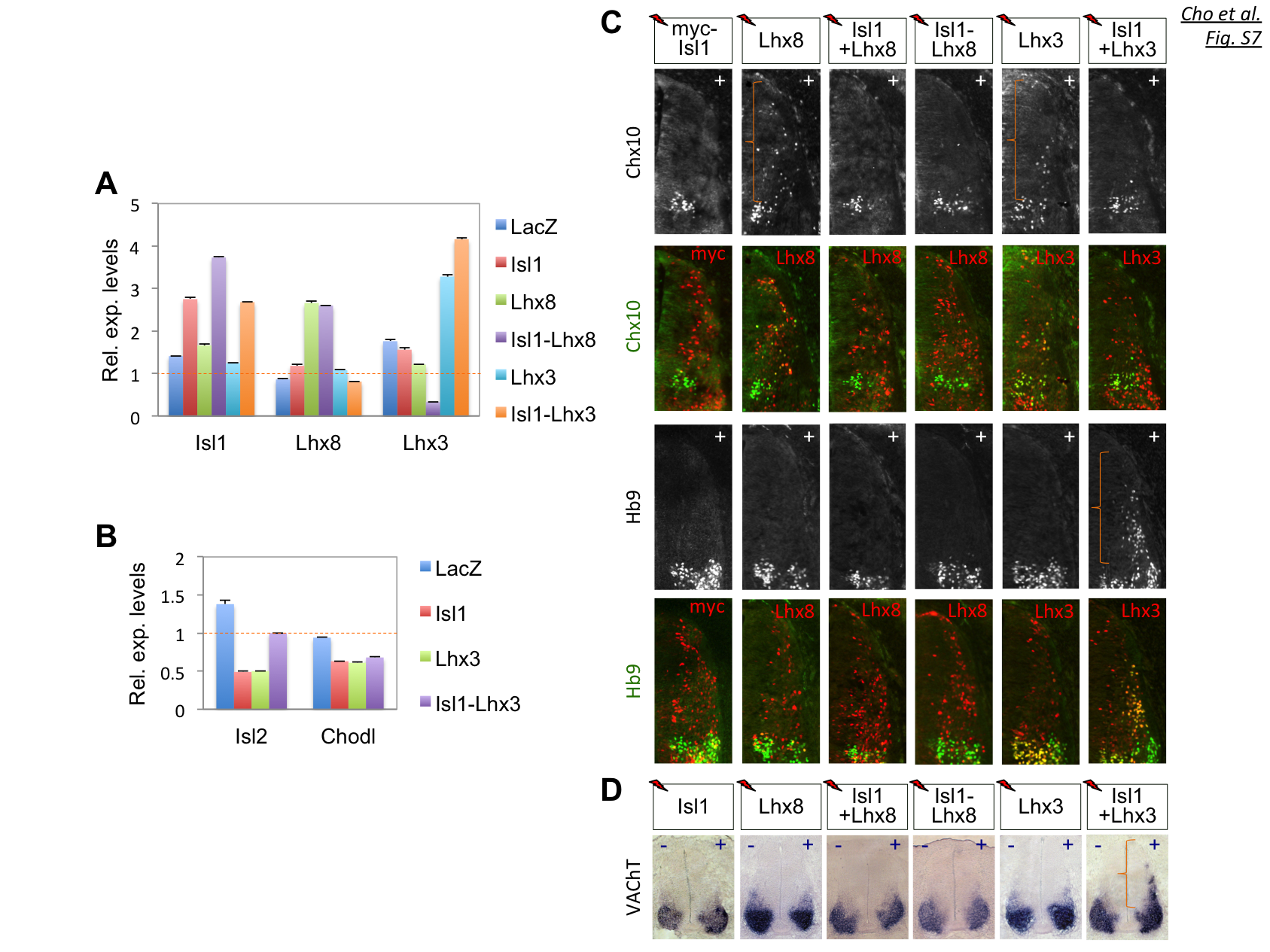

Supplement: Figure S7 — Analyses of mouse or chick embryos electroporated Isl1, Lhx3, Lhx8, Isl1-Lhx3 or Isl1-Lhx8. (A, B) Gene expression analyses in mouse cortices electroporated in utero with constructs, as indicated by color bars. In utero electroporation was performed with E13.5 brains and the qRT-PCR analyses were done in E18.5 cortices. Y-axis indicates the relative expression levels of each gene, shown in the x-axis, on the electropoated side over the control side. The expression from the electroporated constructs was detected by qRT-PCR (A). Expression of Isl1 and Lhx3, either alone or in combination, failed to induce MN genes Isl1 and Chodl. The expression levels of Hb9 were below detection level in qRT-PCR analyses in any of these conditions. Error bars indicate standard deviation. (C, D) Cell differentiation assays in chick embryos electroporated with constructs as indicated on top. Expression of either Lhx8 or Lhx3 led to the ectopic formation of Chx10+ V2 interneurons in the dorsal spinal cord, which was suppressed by co-expression of Isl1. Among all conditions, only co-expression of Isl1 and Lhx3 resulted in ectopic upregulation of Hb9 or VAChT. + indicates the electroporated side. Brackets indicate ectopic Chx10+ V2 interneurons or Hb9+VAChT+ MNs. (TIFF) [file pgen.1004280.s007.tiff]
